# Multi-well capture zones in strip-shaped aquifers

**DOI:** 10.1371/journal.pone.0229767

**Published:** 2020-03-05

**Authors:** Setareh Nagheli, Nozar Samani, D. A. Barry

**Affiliations:** 1 Department of Earth Sciences, Shiraz University, Shiraz, Iran; 2 Laboratoire de technologie écologique (ECOL), Institut des sciences et technologies de l’environnement (IIE), Faculté de l’environnement naturel, architectural et construit (ENAC), Ecole polytechnique fédérale de Lausanne (EPFL), Lausanne, Switzerland; Sapienza Universita di Roma, ITALY

## Abstract

Capture zone equations for a multi-well system in strip-shaped confined and unconfined aquifers with and without regional flow are presented. The aquifer is limited by two parallel boundaries that are either no flow (barrier) or inflow (variable head) so that aquifers with four possible boundary configurations are formed. The wellfield includes any number of extraction or injection wells or a combination of both types. The flow field in the strip-shaped aquifer was converted to its equivalent extensive aquifer using conformal mapping and image well methods. To delineate the capture envelope, the potential, streamline and stagnation point equations were derived using velocity potential theory. The solution permits rapid determination of the effect of number, position and extraction/injection rate of wells, boundary type and direction, and rate of regional flow on the size, shape and pattern of well capture zones. The derived equations are readily extended to water quality and quantity management simulations, as shown by embedding the equations within two optimization schemes, viz., Particle Swarm Optimization (PSO) and Genetic Algorithm (GA), to automatically determine the most efficient wellfield designs for pump-and-treat remediation, contaminant plume containment and pumping policy projects.

## Introduction

Demands on water resources are increasing due to climate-induced changes and population growth [1–3], emphasizing the important role of managing the quantity and quality of water resources [4]. In pumped groundwater systems, knowledge of capture zones is a fundamental element of groundwater management.

The well capture zone within an aquifer is defined as the region from which water is withdrawn by one or more pumping wells [5]. After pumping is initiated, the capture zone grows with time and reaches its maximum size at steady state, which defines the capture envelope [6]. Capture zones are important for aquifer management, groundwater remediation projects [7–10], surface-groundwater interactions, well head protection [11], water rights, and in delineating impacts on transboundary aquifers. For over-exploited aquifers (where annual extraction exceeds recharge), identification of capture zones underpins optimal pumping plans to recover and sustain the depleted storage [12].

Capture zone determination for confined aquifers is long established in groundwater engineering [13–19]. For instance, a two dimensional (2-D) pump-and-treat system was presented by Javandel and Tsang [18] including type curves to determine the number and pumping rate of wells to contain a contamination plume. Three-dimensional (3-D) capture zones are available for both horizontal drains and vertical wells in homogeneous, anisotropic aquifers [20]. Zlotnik [21] studied the effects of anisotropy on capture zones for partially penetrating wells. Analytical and semi-analytical expressions for multiple wells and extraction rates and locations based on complex potential theory and superposition were presented by Christ and Goltz [22]. Their solution was extended by Fienen et al. [23] to the computation of stagnation point locations in a multi-well system. Lu et al. [24] determined stagnation points in a flow field of a single pumping well located at the center of a recharge area.

The above-mentioned studies focused on simple aquifer geometries and did not account for different boundary configurations or unconfined flow. Efforts to extend analytical results include the case of a single well between two parallel streams [5], and multi-well systems in wedge-shaped [6] and peninsula-shaped confined and unconfined aquifers [25]. Capture zones for multi-well systems in rectangular bounded aquifers are also available [26], both for confined and unconfined aquifers. Such analytical solutions are used in optimization schemes. For example, Mantoglou [27] developed analytical models of saltwater intrusion in coastal aquifers of finite size pumped by several wells that were embedded within an optimization algorithm to find the optimal pumping strategy.

The approach most commonly used to obtain analytical results is the image well method [28] in conjunction with complex discharge potential theory. For complex boundary conditions, the number of imaginary wells can be large, and the method can become unwieldy. In this situation, however, conformal mapping can be used, since the number of imaginary wells for individual real single well is then limited to three or less for any aquifer boundary configuration.

Conformal mapping is commonly employed in areas of physics and engineering where Laplace’s equation applies [29–33]. In groundwater flow problems, the modeling of seepage flow was solved by Fukuo and Kaihotsu [34], followed by other applications, e.g., Anderson [35–38]. Fitts [39] obtained an exact solution for two-dimensional flow to a well in an anisotropic domain. Chahar [40] used conformal mapping to provide an analytical solution to the problem of seepage from a soil channel with a curvilinear bottom. Klammler et al. [41] used the conformal mapping method to obtain the solution for flow fields near drain-and-gate reactive barriers. Using the same method, Klammler et al. [42] also developed an approximate analytical solution to the permeable reactive barrier (PRB) capture and release behaviour considering doubly-symmetric funnel-and-gate as well as drain-and-gate configurations. Similarly, Lu et al. [43] derived analytical solutions for pumping in a fully bounded rectangular aquifer. This approach was used by Strack [44] to obtain analytical results for flow to a well between two parallel rivers or rivers that are at a fixed angle. These results are generalized here.

Our purpose is to obtain a general solution for the capture zone of a multi-well system in bounded strip-shaped aquifers with or without uniform regional flow. The well system includes any number of arbitrarily located extraction or injection wells. The strip-shaped aquifer is located between parallel inflow (variable head) boundaries (surface water bodies like streams, rivers, canals, lakes, and seas) and no-flow (barrier) boundaries (bedrock, fault blocks, glacial tills or impervious rocks). Conformal mapping is applied to determine the complex discharge potential and streamline equations for determining the capture envelope. The method permits efficient and rapid modeling of capture envelopes, as shown by examples where the effects of number, position and extraction rate of wells and regional flow direction and rate on the capture envelope are investigated. We demonstrate the versatility of the approach by its application to groundwater quantity and quality management problems. Specifically, the capture zone model is embedded within a Particle Swarm Optimization (PSO) [45] and Genetic Algorithm (GA) [46] schemes to optimize design of pump-and-treat remediation, contaminant plume containment and pumping projects.

## Conceptual model

**Fig 1:**
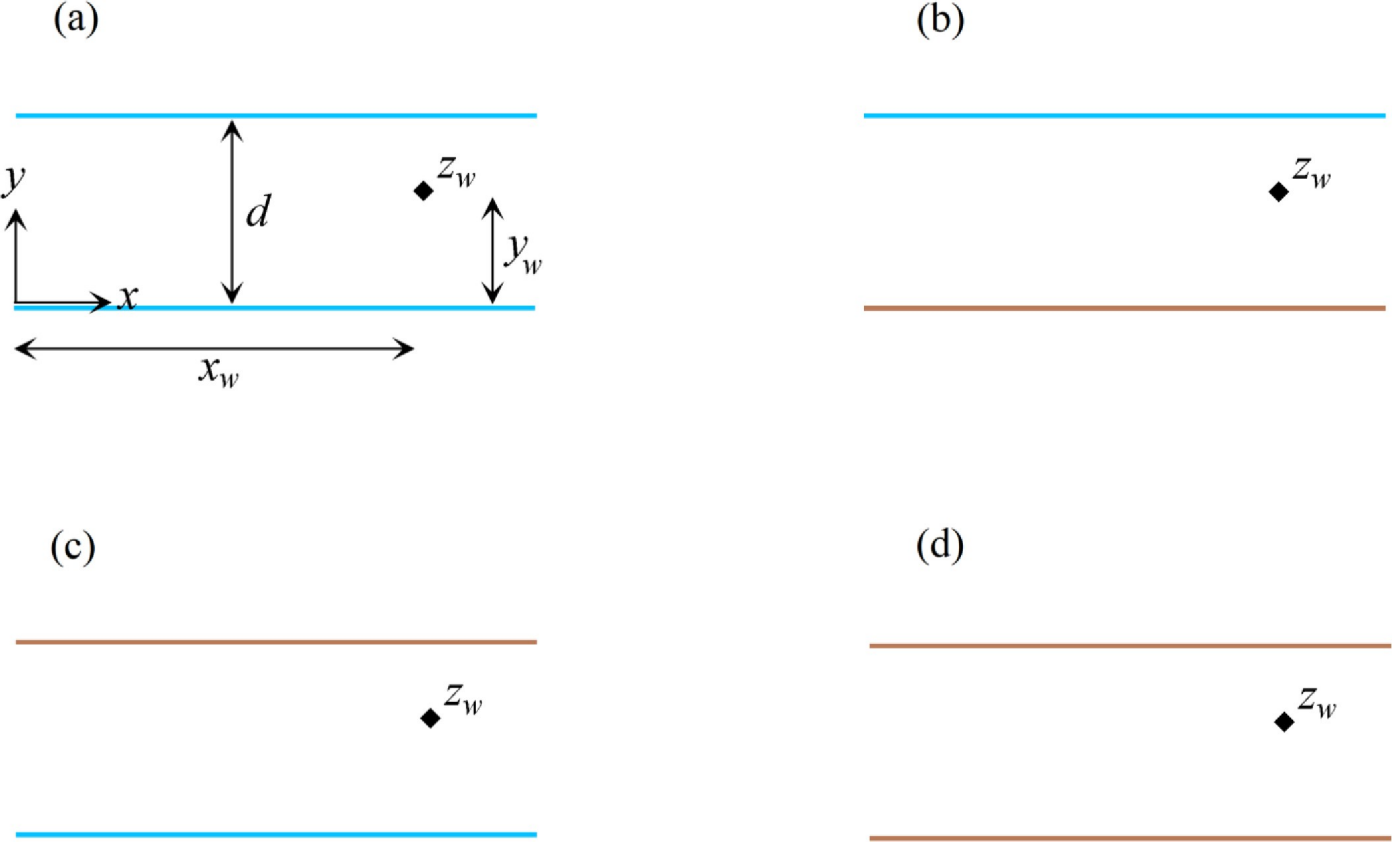
**Schematic plan view of a strip-shaped aquifer with various boundary configurations: a) inflow-inflow, b) inflow-barrier, c) barrier-inflow and d) barrier-barrier.** In this figure, blue and brown lines show inflow and barrier boundaries, respectively. Black diamonds represent wells.

Fig 1 shows a schematic plan view of different aquifer (confined or unconfined) configurations. Each case shows an aquifer with a fully penetrating well bounded by two parallel boundaries of infinite extent forming a strip-shaped aquifer. The aquifer is isotropic and homogeneous with uniform thickness. Steady state, 2-D flow is considered. In Fig 1A, the boundaries are fully penetrating streams (variable head boundary) having no hydraulic resistance with the aquifer. An extraction or injection well is located at (*x*_w_,*y*_w_). Note that the solution is for a multi-well system with arbitrary extraction/injection rates, and steady regional flow at an arbitrary angle (*β*) relative to positive *x*-axis. On physical grounds, the flow direction must be consistent with the hydraulic properties of the boundaries in that if the direction of regional flow is perpendicular to streams (*β* = *π*/2 or 3*π*/2), they are treated as constant head boundaries. In Fig 1B and 1C, one boundary is an inflow stream (variable head boundary) and another one is an impervious (no-flow) boundary. The aquifer depicted in Fig 1C is different from that of Fig 1B due to the position of wells relative to boundaries and vice versa. As a result, they should be treated as two separate well-aquifer systems. There are two no-flow boundaries in Fig 1D. In this case, on physical grounds, the regional flow direction must be parallel to the impermeable boundaries.

## Mathematical formulation

### Capture zone

**Fig 2:**
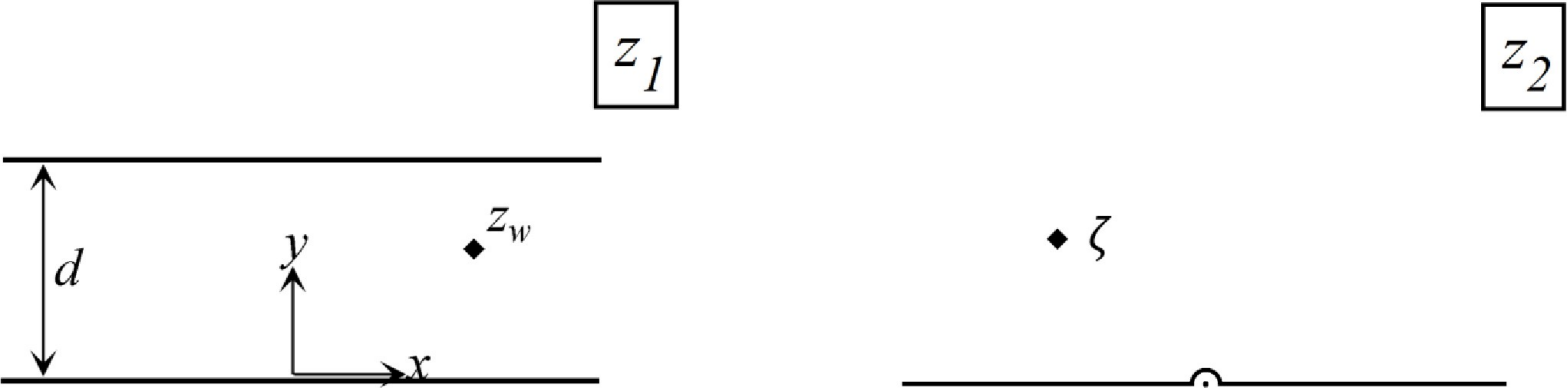
Mapping the conceptual model from the physical plane (*z*_1_) to the conformal mapping plane (*z*_2_).

**Fig 3:**
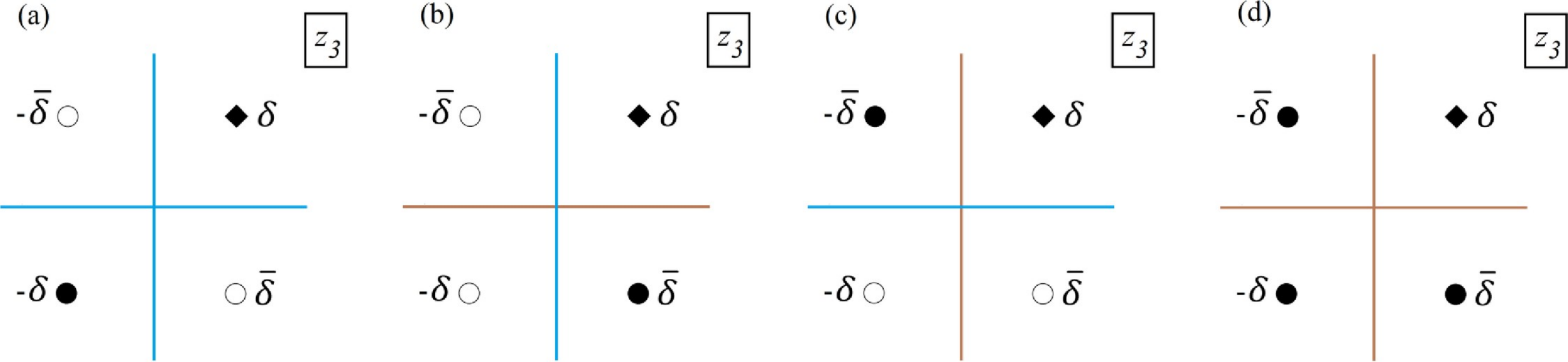
The conceptual model in the *z*_3_−*plane*. Solid diamonds are locations of the real wells. Solid circles are the image wells of the same type as the real well (i.e., injection/extraction) and the hollow circles denote image wells of the opposite type to the real well. *δ* shows the spatial location of the extraction or injection well in the *z*_3_−*plane* and $\bar{\delta }$ is complex conjugate of *δ*.

Using conformal mapping, the conceptual model of Fig 1 (*z*_1_−*plane*) is first mapped onto the upper half-plane in the *z*_2_−*plane* (Fig 2) using *z*_2_ = exp(*πz*_1_/*d*) (see S1 File for details). When the boundaries are a combination of barrier-inflow and inflow-barrier, the *z*_1_−*plane* is transformed into the second auxiliary plane, the *z*_3_−*plane*, using the transformation $z_{3}={z_{2}}^{1/2}$. Transforming the zones in Fig 1A–1D to the *z*_3_−*plane* is not necessary but generalizes the equation of capture zones. The four aquifers of Fig 1 were transformed to the *z*_3_−*plane* as shown in Fig 3. Transformation from the *z*_1_−*plane* to the *z*_3_−*plane* is given by:

$$z_{3}=\exp\left(\frac{\pi z_{1}}{2d}\right)$$ (1)

The complex potential for flow towards a single well at position *ζ* in the *z*_2_−*plane* with the regional uniform flow in an aquifer bounded by two parallel streams is [44]:

$$\Omega (z_{2})=\phi_{0}-\frac{dq_{0}}{\pi }\ln\left(z_{2}\right)\exp\left(-i\beta \right)+\frac{Q_{w}}{2\pi }\ln\left(\frac{z_{2}-\zeta }{z_{2}-\bar{\zeta }}\right)$$ (2)

where, *Q*_*w*_ is the pumping or injection rate (L^3^T^-1^), *q*_0_ is the uniform flow rate in the direction *β* (radians) relative to the positive *x* axis, *ϕ*_0_ is the arbitrary potential in the absence of extraction and regional flow. Writing Eq (2) in the *z*_3_−*plane* gives:

$$\Omega (z_{3})=\phi_{0}-\frac{2dq_{0}}{\pi }\ln\left(z_{3}\right)\exp\left(-i\beta \right)+c\frac{Q_{w}}{2\pi }ln\left[\frac{\left(z_{3}-\delta )(z_{3}+\delta \right)}{\left(z_{3}-\bar{\delta })(z_{3}+\bar{\delta }\right)}\right]$$ (3)

The parameter *c* is +1 for extraction wells and -1 for injection wells, respectively. Generalizing Eq (3) for the four boundary configurations of Fig 3 results in:

$$\Omega (z_{3})=\phi_{0}-\frac{2dq_{0}}{\pi }\ln\left(z_{3}\right)\exp\left(-i\beta \right)+c\frac{Q_{w}}{2\pi }[J_{1}\ln\left(z_{3}-\delta \right)+J_{2}\ln(z_{3}+\delta )+J_{3}\ln(z_{3}-\bar{\delta })+J_{4}\ln\left(z_{3}+\bar{\delta }\right)]$$ (4)

where parameters *J*_1_ to *J*_4_ take values as given in Table 1.

**Table 1:** Values of parameters *J*_1_ to *J*_4_ in Eq (8) for various boundary configurations.

Boundary configuration type	Boundary configuration	*J*_1_	*J*_2_	*J*_3_	*J*_4_
**a**	Constant head-Constant head	+1	+1	-1	-1
**b**	Constant head-No flow	+1	-1	+1	-1
**c**	No flow-Constant head	+1	-1	-1	+1
**d**	No flow-No flow	+1	+1	+1	+1

Because of the linearity of Laplace’s equation, Eq (3) may be extended for a multi-well system using the principle of superposition:

$$\Omega (z_{3})=\phi_{0}-\frac{2dq_{0}}{\pi }\ln\left(z_{3}\right)\exp\left(-i\beta \right)+\sum_{j=1}^{N}c_{j}\frac{Q_{wj}}{2\pi }[J_{1}\ln\left(z_{3}-\delta_{j}\right)+J_{2}\ln(z_{3}+\delta_{j})+J_{3}\ln(z_{3}-{\bar{\delta }}_{j})+J_{4}\ln\left(z_{3}+{\bar{\delta }}_{j}\right)]$$ (5)

where *N* is the number of wells. The substitution of *z*_3_ for *z*_1_ gives:

$$\Omega (z_{1}){=\phi }_{0}-q_{0}z_{1}\;\exp\left(-i\beta \right)+\sum_{j=1}^{N}c_{j}\frac{Q_{wj}}{2\pi }\{J_{1}\ln\left[exp(\frac{\pi z_{1}}{2d})-exp(\frac{\pi z_{wj}}{2d})\right]+J_{2}\ln\left[exp(\frac{\pi z_{1}}{2d})+exp(\frac{\pi z_{wj}}{2d})\right]+J_{3}\ln\left[exp(\frac{\pi z_{1}}{2d})-exp(\frac{\pi {\bar{z}}_{wj}}{2d})\right]+J_{4}\ln\left[exp(\frac{\pi z_{1}}{2d})+exp(\frac{\pi {\bar{z}}_{wj}}{2d})\right]\}$$ (6)

Using the following dimensionless quantities:

$$\begin{array}{l} z_{1D}=\frac{z_{1}}{d}\;z_{wDj}=\frac{z_{wj}}{d}\;Q_{wDj}=\frac{Q_{wj}}{Kbd}\;q_{0D}=\frac{q_{0}}{Kb}\\ \Omega_{D}=\frac{\Omega }{Kbd}\;\phi_{D}=\frac{\phi }{Kbd}\;\psi_{D}=\frac{\psi }{Kbd} \end{array}$$ (7)

Eq (6) becomes:

$$\Omega_{D}=\phi_{0D}-q_{0D}(x_{D}+iy_{D})exp(-i\beta )+\sum_{j=1}^{N}c_{j}\frac{Q_{wDj}}{2\pi }\left[J_{1}\ln\left(f_{1Dj}+if_{2Dj}\right)+J_{2}\ln\left(g_{1Dj}+ig_{2Dj}\right)+J_{3}\ln\left(f_{1Dj}+ig_{2Dj}\right)+J_{4}\ln\left(g_{1Dj}+if_{2Dj}\right)\right]$$ (8)

with *f*_1*Dj*_, *f*_2*Dj*_, *g*_1*Dj*_ and *g*_2*Dj*_ as given in Appendix A. The real part of Eq (8) gives the dimensionless potential, *ϕ*_*D*_:

$$\phi_{D}=\phi_{0D}-q_{0D}{(x}_{D}\cos\beta {+y}_{D}\sin\beta )+\sum_{j=1}^{N}c_{j}\frac{Q_{wDj}}{4\pi }\left[J_{1}\ln\left({f_{1Dj}}^{2}+{f_{2Dj}}^{2}\right)+J_{2}\ln\left({g_{1Dj}}^{2}+{g_{2Dj}}^{2}\right)+J_{3}\ln\left({f_{1Dj}}^{2}+{g_{2Dj}}^{2}\right)+J_{4}\ln\left({g_{1Dj}}^{2}+{f_{2Dj}}^{2}\right)\right]$$ (9)

while the imaginary part gives the dimensionless stream function, *ψ*_*D*_:

$$\psi_{D}=-q_{0D}(y_{D}\;\cos\beta -x_{D}\sin\beta )+\sum_{j=1}^{N}c_{j}\frac{Q_{wDj}}{2\pi }[J_{1}{tan}^{-1}(\frac{f_{2Dj}}{f_{1Dj}})+J_{2}{tan}^{-1}(\frac{g_{2Dj}}{g_{1Dj}})+J_{3}{tan}^{-1}(\frac{g_{2Dj}}{f_{1Dj}})+J_{4}{tan}^{-1}(\frac{f_{2Dj}}{g_{1Dj}})]$$ (10)

Plotting Eqs (9) and (10) results in a flow net that illustrates the capture curves of a multi-well system in a strip-shaped aquifer in dimensionless form. The flow net is conveniently plotted using the contour syntax in MATLAB. The stream function has a branch cut for each well that may be removed with the unwrap syntax in MATLAB.

In unconfined aquifers, the saturated thickness varies, so the potential equals to:

$$\phi =\frac{1}{2}Kh^{2}$$ (11)

Eqs (8–10) can be rewritten for unconfined aquifer by replacing the product *Kb* with $K{\bar{h}}_{0}$ (discharge per unit width) and using the following dimensionless terms:

$$Q_{wDk}=\frac{Q_{wk}}{K{\bar{h}}_{0}d}\;q_{0D}=\frac{q_{0}}{K{\bar{h}}_{0}}\;\Omega_{D}=\frac{\Omega }{K{\bar{h}}_{0}d}\;\phi_{D}=\frac{\phi }{K{\bar{h}}_{0}d}\;\psi_{D}=\frac{\psi }{K{\bar{h}}_{0}d}$$ (12)

### Stagnation points

The capture envelopes that pass through the stagnation points (where the flow velocity is zero) in the flow field separate the regions of flow. To find the position of stagnation points, the derivative of the complex potential (Eq 5) is taken with respect to *z*_3_ and the result set equal to zero:

$$\frac{d\Omega (z_{3})}{dz_{3}}=-\frac{{2dq}_{0}}{\pi z_{3}}exp(-i\beta )+\sum_{j=1}^{N}c_{j}\frac{Q_{wj}}{2\pi }[\frac{J_{1}}{z_{3}-\delta_{j}}+\frac{J_{2}}{z_{3}+\delta_{j}}+\frac{J_{3}}{z_{3}-{\bar{\delta }}_{j}}+\frac{J_{4}}{z_{3}+{\bar{\delta }}_{j}}]=0$$ (13)

The roots of Eq (13) are the locations of stagnation points in the *z*_3_−*plane*, which are easily transformed to the *z*_1_−*plane*. Eq (13) was solved using MATLAB. The values of the stream function at the stagnation points are calculated (Eq 10) and the capture envelopes are drawn by the stream line passing through the stagnation points.

### Drawdown

The steady state drawdown (*s*) in a confined aquifer from a fully penetrating extraction well is [47]:

$$s=h_{0}-h=\frac{Q_{w}}{2\pi Kb}ln\left(\frac{r_{0}}{r}\right)$$ (14)

where *h*_0_ and *h* are hydraulic heads at distance *r*_0_ and *r* from the well, respectively. Since the velocity potential is *ϕ* = *Kh* and the dimensionless head is *h*_*D*_ = *h*/*d*, the dimensionless drawdown (*s*_*D*_) can be written in terms of dimensionless potential as:

$$s_{D}=\phi_{0D}-\phi_{D}$$ (15)

where potentials *ϕ*_0*D*_ and *ϕ*_*D*_ are calculated using Eq (9). The steady state drawdown (*s*) in an unconfined aquifer at a distance (*r*) from a fully penetrated extraction well with radius (*r*_*w*_) is [47]:

$$h_{0}^{2}-h^{2}=\frac{Q_{w}}{\pi K}ln\left(\frac{r_{0}}{r}\right)$$ (16)

Using Eq (11) the dimensionless head in an unconfined is written as:

$$h_{0}^{2}=\frac{2\phi_{0D}{\bar{h}}_{0}}{d},\;h^{2}=\frac{2\phi_{D}{\bar{h}}_{0}}{d}$$ (17)

From Eqs (16) and (17), the dimensionless drawdown in an unconfined aquifer is:

$$s_{D}={\left(\frac{2\phi_{0D}{\bar{h}}_{0}}{d}\right)}^{\frac{1}{2}}-{\left(\frac{2\phi_{D}{\bar{h}}_{0}}{d}\right)}^{\frac{1}{2}}$$ (18)

Eqs (15) and (18) can be used to demonstrate the application of proposed model to water quantity management (see Water quantity management projects Section below).

## Results and Discussion

In the following sections, Eqs (9), (10) and (13) are solved for strip-shaped aquifers with different boundary configurations and flow conditions, after which we plot the capture envelopes of the well(s). First, the effects of various boundary configurations (Fig 1) on the shape and properties of the capture zones are investigated, then the effect of extraction rates and regional flow direction and rate are explored. Due to numerous parameters (number, type, location and extraction/injection rates of wells, and the rate and direction of regional flow) that influence the flow field, many capture envelopes can be simulated. Here, representative examples of capture zones in each parallel boundary configuration are presented, along with the water quantity management and optimized remediation schemes for a pump-and-treat method.

### Effects of boundary configuration on the capture zones

#### Capture zone of well(s) in a strip-shaped aquifer with inflow-inflow boundaries

**Fig 4:**
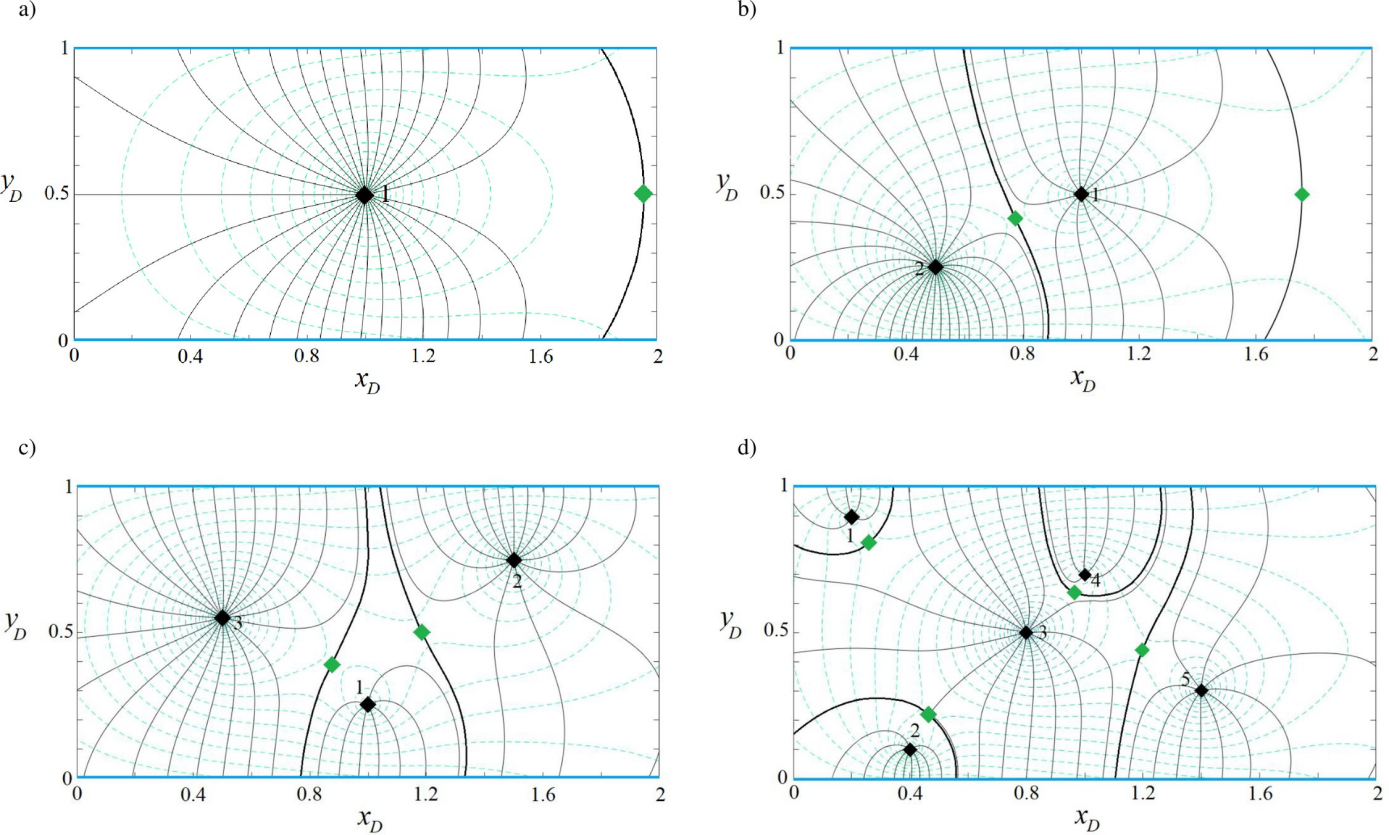
**Velocity potential (dashed lines) and stream function (solid grey lines) for one (a), two (b), three (c) and five (d) wells in a strip-shaped aquifer with inflow-inflow boundaries.** In this figure and Figs 5–7: *β* = 0. Thick black curves show the limit of the capture envelopes and black and green diamonds represent wells and stagnation points, respectively.

In this example, we consider a strip-shaped aquifer bounded with inflow-inflow boundaries (Fig 1A) in which the direction *β* and the rate *q*_0*D*_ of the uniform regional flow are 0 rad and 0.001, respectively. The parameter *J* is defined in Table 1. Eqs (9), (10), and (13) are solved for 1, 2, 3 and 5 wells (the position and pumping rate of extraction wells are defined in Table 2). Fig 4A–4D illustrate, respectively for the four cases, their potential, streamlines, stagnation points and capture envelopes. The black diamond shows the well position and the green diamond illustrates the position of the stagnation point. The dashed lines indicate the velocity potential and the solid lines represent the streamline reaching the location of the extraction well. In Fig 4A, the well extraction rate is provided from regional flow and two inflow boundaries and the capture envelope extends symmetrically. Due to the direction of regional flow, the capture envelope trends toward the west (left) side. In Fig 4B, well (2) extracts water from the streams as well as the uniform regional flow. A sharp groundwater divide, effectively a barrier, is established between two wells so that the uniform regional flow provides no water to well (1). Streamlines run parallel along the divide. In Fig 4C, well (3) has the largest capture envelope because it has the maximum extraction rate. The three wells gain water from both boundaries. Two groundwater divides are also formed, which separates the capture zone of well (2) from those of the other wells. In Fig 4D, each well has its own capture envelope. Wells (1) and (2) gain water from the north and south side boundaries, respectively, whereas well (3) is recharged by the two boundaries and regional flow. Its capture zone shape shows the influence of the other wells. Well (4) is fully supplied by the northern boundary. A groundwater divide separates the capture zone of well (5) from those of the others while both boundaries supply water to it. In both Fig 4C and 4D, a stagnation point exists for wells (2) and (5) that falls at the far right side out of the plotted capture zones. Note that in Fig 4 and other figures regarding the capture zones, the branch cut is removed. However, as an example Fig 4 with branch cuts is given in Figure B in S1 File.

**Table 2:** Extraction rates and coordinates of extraction wells in Figs 4–7.

*Figs 4–7*	*Well number*	*x*_*wD*_	*y*_*wD*_	*Q*_*wD*_
**a**	1	1	0.5	0.02
**b**	1	1	0.5	0.02
	2	0.5	0.25	0.04
**c**	1	1	0.25	0.02
	2	1.5	0.75	0.04
	3	0.5	0.55	0.06
**d**	1	0.2	0.9	0.02
	2	0.4	0.1	0.04
	3	0.8	0.5	0.06
	4	1	0.7	0.02
	5	1.4	0.3	0.04

The dimensionless values of parameters (i.e., ***x***_***wD***_, ***y***_***wD***_, ***Q***_***wD***_) can be calculated using Eq (6).

#### Capture zone of well(s) in a strip-shaped aquifer with inflow-barrier boundary conditions

**Fig 5:**
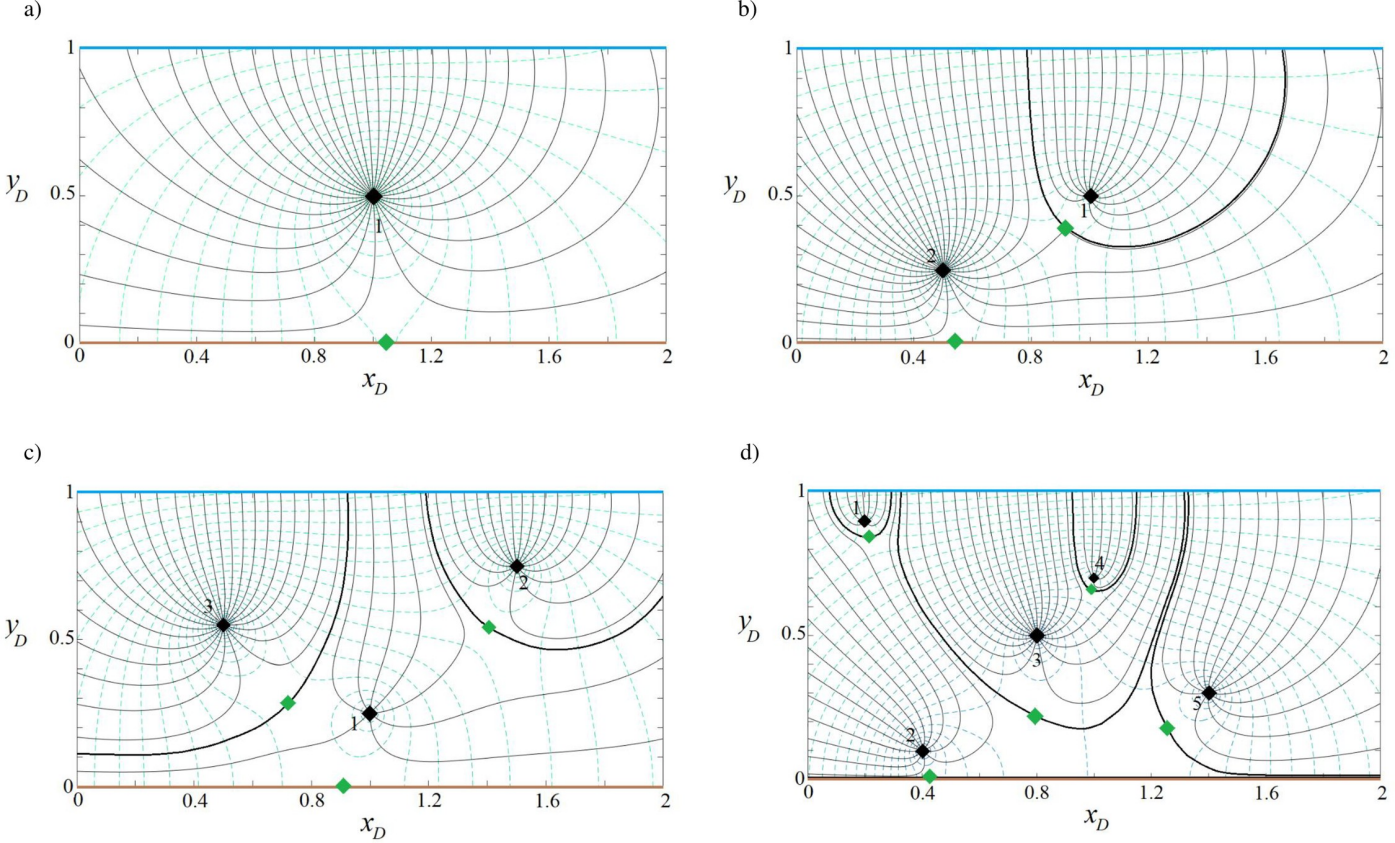
Velocity potential and stream function for one (a), two (b), three (c) and five (d) wells in a strip-shaped aquifer with inflow-barrier boundary conditions.

For this boundary configuration (Fig 1B), the aquifer thickness, hydraulic conductivity and the direction and regional flow rate are the same as the previous examples, as specified in Table 2 with *J* values as given in Table 1. Eqs (9), (10) and (13) are solved for 1, 2, 3 and 5 wells and the resulting capture curves are plotted in Fig 5. In Fig 5A, the case of 1 well, the extracted water is provided from both the regional flow and the inflow boundary. The streamlines are parallel to the no-flow boundary as expected. In Fig 5B, well (1) is mainly supplied by the stream, while well (2) gains water from regional flow and the stream. In Fig 5C, well (3) has the largest extraction rate, with water supplied by the stream and the regional flow. A small portion of regional flow reaches well (1). The capture zone of well (1) extends towards the stream as well as the west and east sides of the aquifer. The capture envelope of well (2) is smaller, due to its short distance from the north boundary and lower extraction rate. Fig 5D demonstrates the competition among wells in gaining water from the north boundary based on their pumping rate and distance from the boundaries. Only well (2) benefits from regional flow and its capture zone is limited by the no-flow boundary and manages to gain a small portion of water from the stream along three narrow zones: a) between the capture envelopes of wells (3) and (5), b) the capture envelopes of wells (1) and (3) and c) the capture zone of well (5) and the no-flow boundary.

#### Capture zone of well(s) in a strip-shaped aquifer with barrier-inflow boundaries

**Fig 6:**
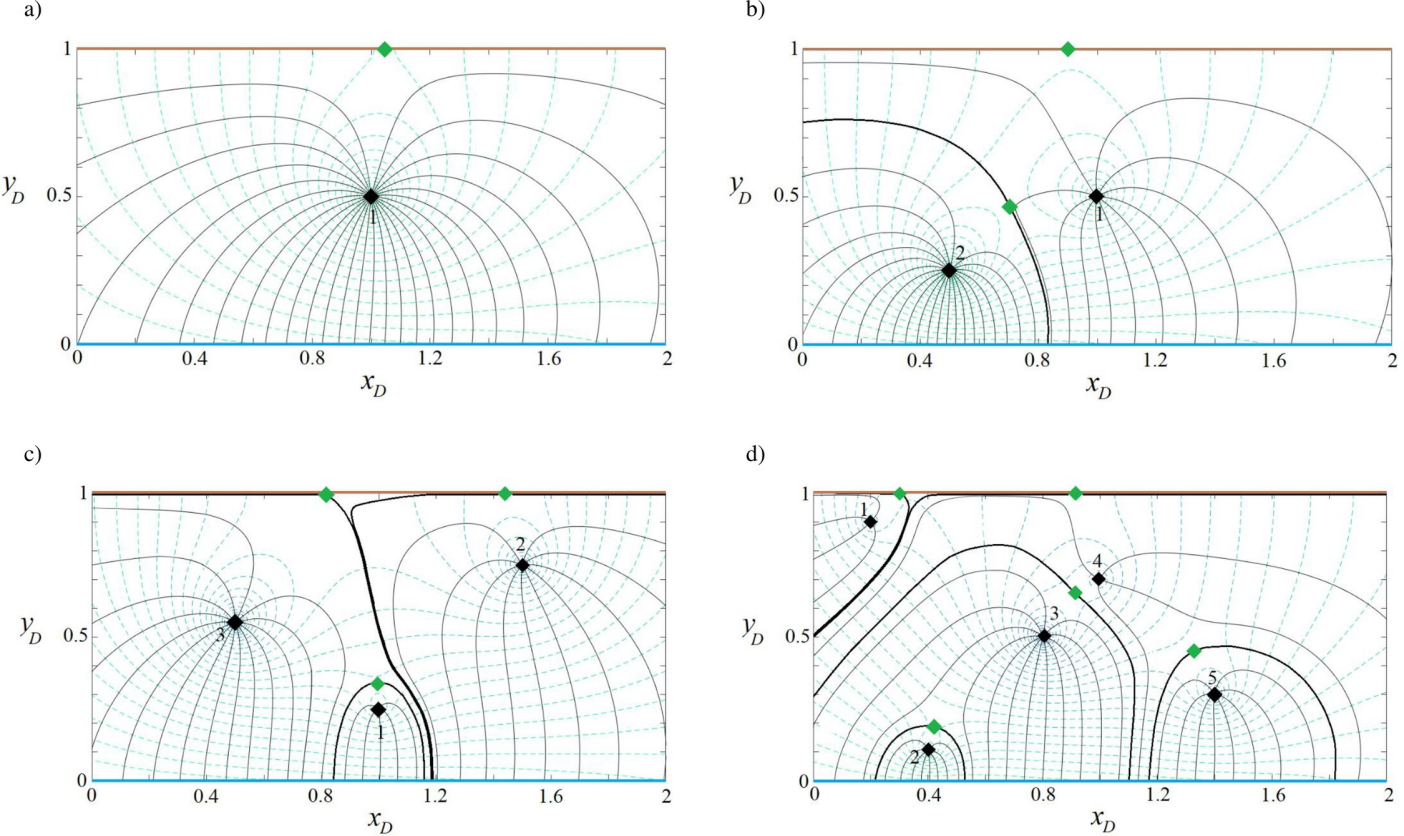
Velocity potential and stream function for one (a), two (b), three (c) and five (d) wells in a strip-shaped aquifer with barrier-inflow boundary conditions.

Fig 6 shows results computed using Eqs (9), (10) and (13), solved for the configuration shown in Fig 1C, with parameters as given in Tables 1 and 2. The aquifer thickness, hydraulic conductivity and direction and rate of regional flow are the same as in the previous examples. Fig 6A depicts that well (1) is supplied by the stream as well as regional flow. In Fig 6B, wells (1) and (2) gain water from the stream and well (2) captures the main portion of regional flow and allows a smaller portion to reach well (1). In Fig 6C, the stagnation points of wells (2) and (3) are positioned on the barrier boundary and form a small stagnation zone. Well (3) prevents regional flow to reach wells (1) and (2) and encompasses the capture envelope of well (1). As a result, wells (2) and (1) are only fed by the inflow boundary. Fig 6D demonstrates that well (1) is recharged by regional flow only and wells (2) and (5) by the stream. Water reaches well (4) via a narrow pathway connecting the well to the upstream regional flow and via two sections of the inflow boundary. Well (3) is fed by the stream and its capture envelope encompasses that of well (2).

#### Capture zone of well(s) in a strip-shaped aquifer with barrier-barrier boundaries

**Fig 7:**
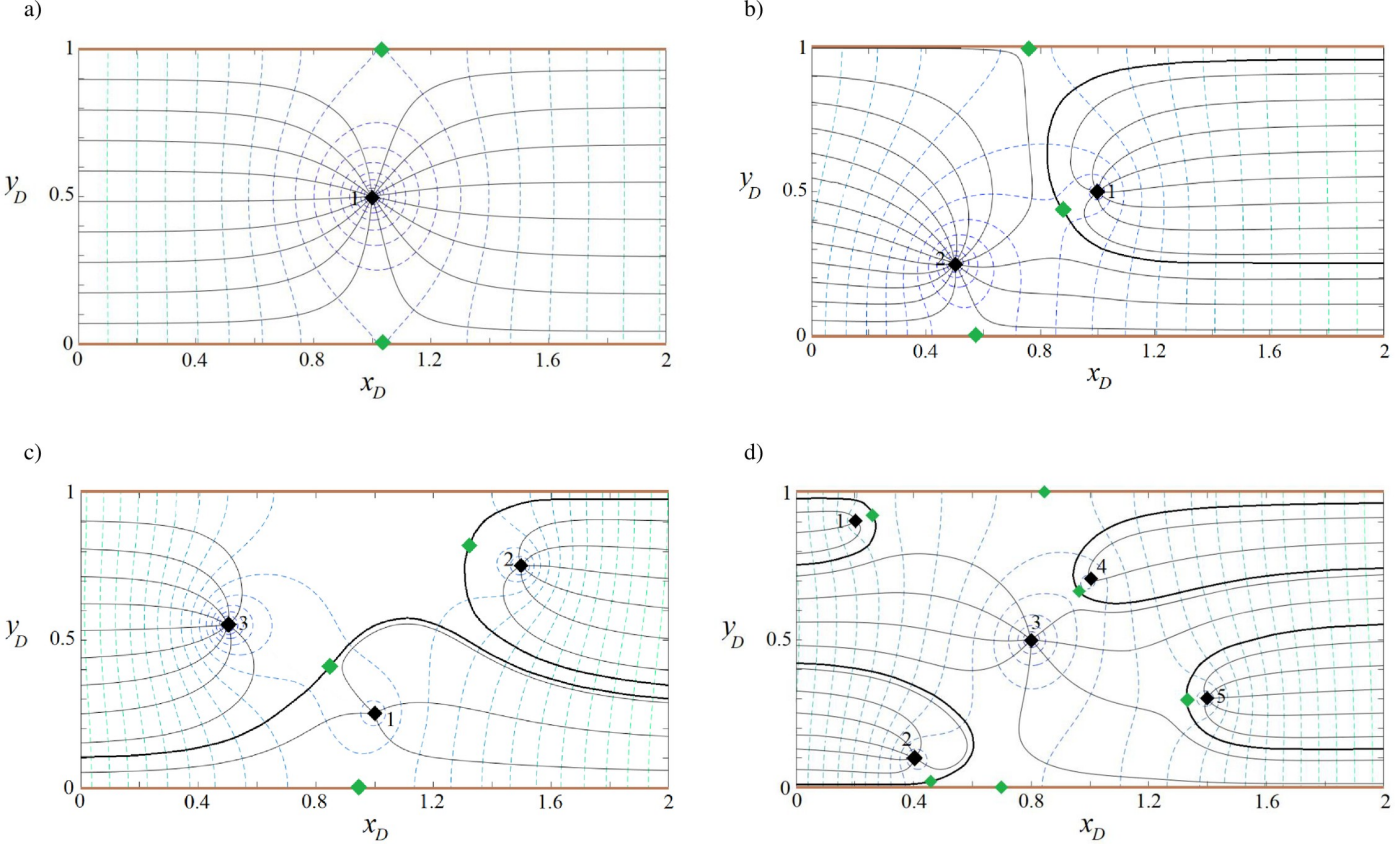
Velocity potential and stream function for one (a), two (b), three (c) and five (d) wells in a strip-shaped aquifer with barrier-barrier boundary conditions.

The aquifer in this example corresponds to the case in previous section. The difference is that the in-flow boundary is replaced by barriers (Fig 1D). According to Table 1, for this boundary configuration the value of parameters *J*_1_, *J*_2_, *J*_3_ and *J*_4_ are all equal to +1. As above, the regional flow direction is from west to east (*β* = 0). Eqs (9), (10) and (13) are solved for 1, 2, 3 and 5 well systems–potential and streamline functions are plotted in Fig 7. As shown in the figure, the streamlines are parallel to the barrier boundary before merging to the wells and equipotential lines are perpendicular to the barrier boundaries, as expected. In Fig 7A, the extraction rate is provided by regional flow from the west side of the aquifer and also from the infinite boundary in the east. As a result, the west and east flowing fronts intersect and two stagnation points form on the boundaries. In Fig 7B, regional flow is fully captured by well (2) and well (1) gains water from the east infinite side. In Fig 7C, the capture envelopes of well (3) and well (2) extend to the west and east sides, respectively, while that of well (1) extends to both sides along two narrow channels parallel to the barrier boundary. In Fig 7D wells (1) and (2) capture water from the west side of the aquifer and wells (4) and (5) from the east side while well (3) captures water from both sides. Well (3) forms two stagnation points each located on one barrier boundary. Comparing this figure with Figs 4–6, one can see how boundary types, pumping rates, and well(s) position control the shape, size and pattern of capture envelopes. Wells interact differently with each other and with the boundaries. In a regional pumping scheme, these differences are important for water quantity management, allocation of water rights, and for pumping rate permits.

### Effects of regional flow direction and rate on the capture zones

**Fig 8:**
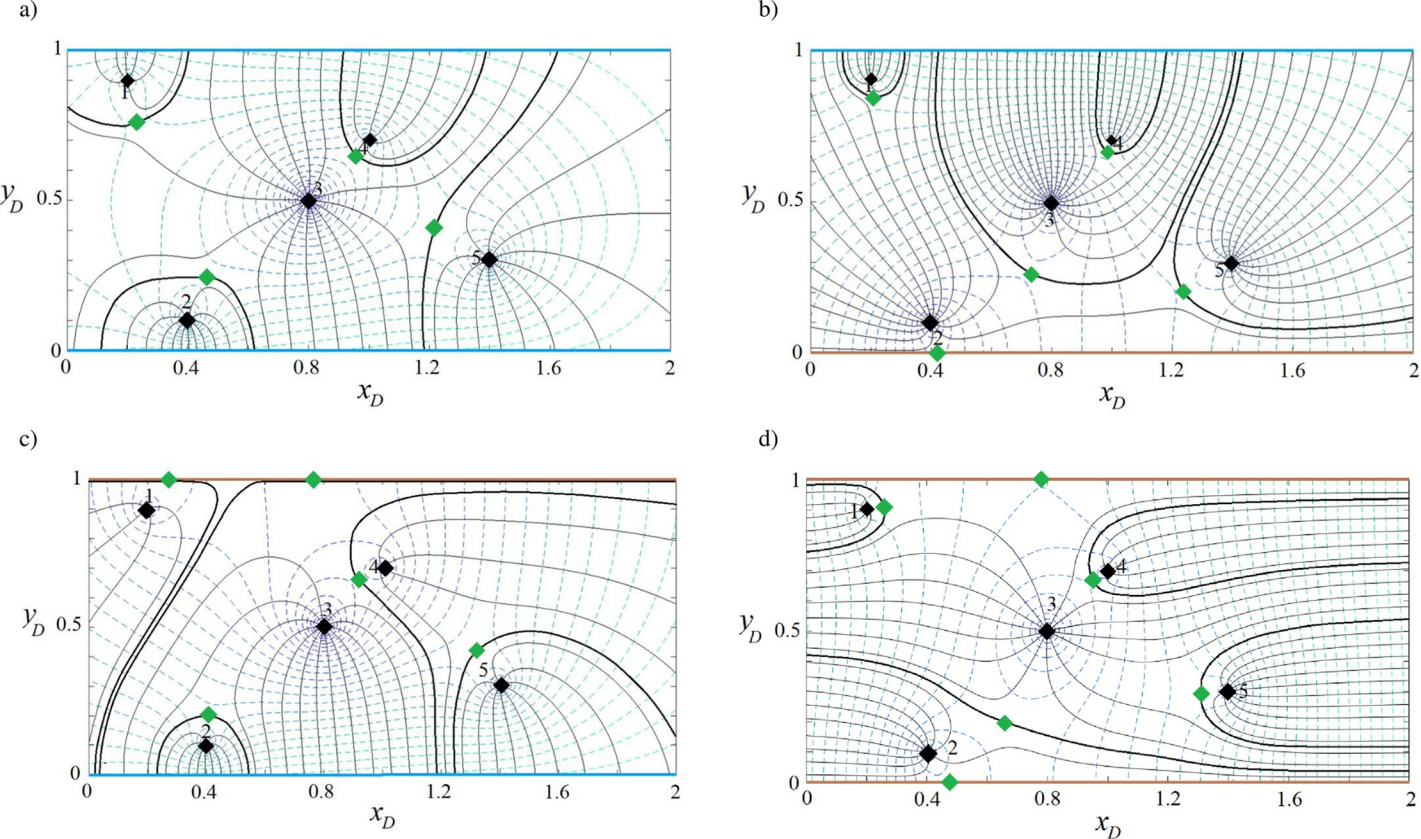
Velocity potential and streamlines when *β* = *π* in a strip-shaped aquifer with five extraction wells.

In the previous examples, we assumed that the direction of regional flow was from west to east (*β* = 0). To demonstrate the effect of regional flow direction on the capture envelopes, Figs 4D, 5D, 6D and 7D are replotted for *β* = *π* in Fig 8 while Fig 4B and 4D are replotted for *β* = *π*/2 in Figure C in S1 File. The difference due to the flow direction is profound, as readily seen in the shape, size and pattern of capture envelopes and the interaction of wells and boundaries. In Fig 8A, in contrast to its corresponding Fig 4D, well (5) fully captures and prevents regional flow to reach other wells. As the result, it is fed along a shorter segment of the north boundary; the capture zone of well (1) leans somewhat to the west and its stagnation point is displaced further south; well (2) is only fed by the southern stream; well (3) is fed along a longer section of the northern stream and a shorter segment of the southern stream while the capture envelope of well (4) slopes further to the east. In Fig 8B, contrary to Fig 5D, regional flow is mainly captured by well (5) although and a small portion of it is captured by well (2) through a narrow flow channel along the no-flow boundary. In Fig 8C, in comparison to Fig 6D, the pattern of capture envelopes changes considerably and regional flow is captured by wells (4) and (3) instead; well (1) reaches the stream and gain water from it; the capture envelope of well (4) encompasses that of well (5), which moves further to the east. In Fig 8D, wells (2–5) instead of wells (1–3) in Fig 7D capture regional flow. In Figure Ca in S1 File, in comparison to Fig 4B, regional flow is captured by both wells, the capture envelope of well (2) extends further to the east and its stagnation point moves up close to the north boundary. In Figure Cb in S1 File, compared to Fig 4D, regional flow is captured by wells (2), (3) and (5); the capture envelopes greatly change in size and shape and pattern; the capture envelope of well (1) becomes larger than that of well (3), which extends to the east by encompassing that of well (5); well (2) is only supplied by the southern boundary.

The dimensionless regional flow is increased to 0.02 compared to that of the cases presented in Figs 4–8 (which was 0.001), with the new results given in Figures D-H in S1 File. All the capture envelopes in these figures extend in the opposite sense to the regional flow direction, i.e., the regional flow contributes to the extraction rate of all wells. It is clear that the size, shape, dimensions and pattern of well capture zones and the interaction of boundaries and wells are greatly changed compared to Figs 4–8.

Figs 4–8 and Figures C-H in S1 File demonstrate that the capture zone model developed above can help delineate the interaction between surface-subsurface flows in water resources management projects such as allocating water rights, issuing well drilling permits, pumping rate permits, etc., and for the sustainable development of water resources in a region.

### Validation of the solution

**Fig 9:**
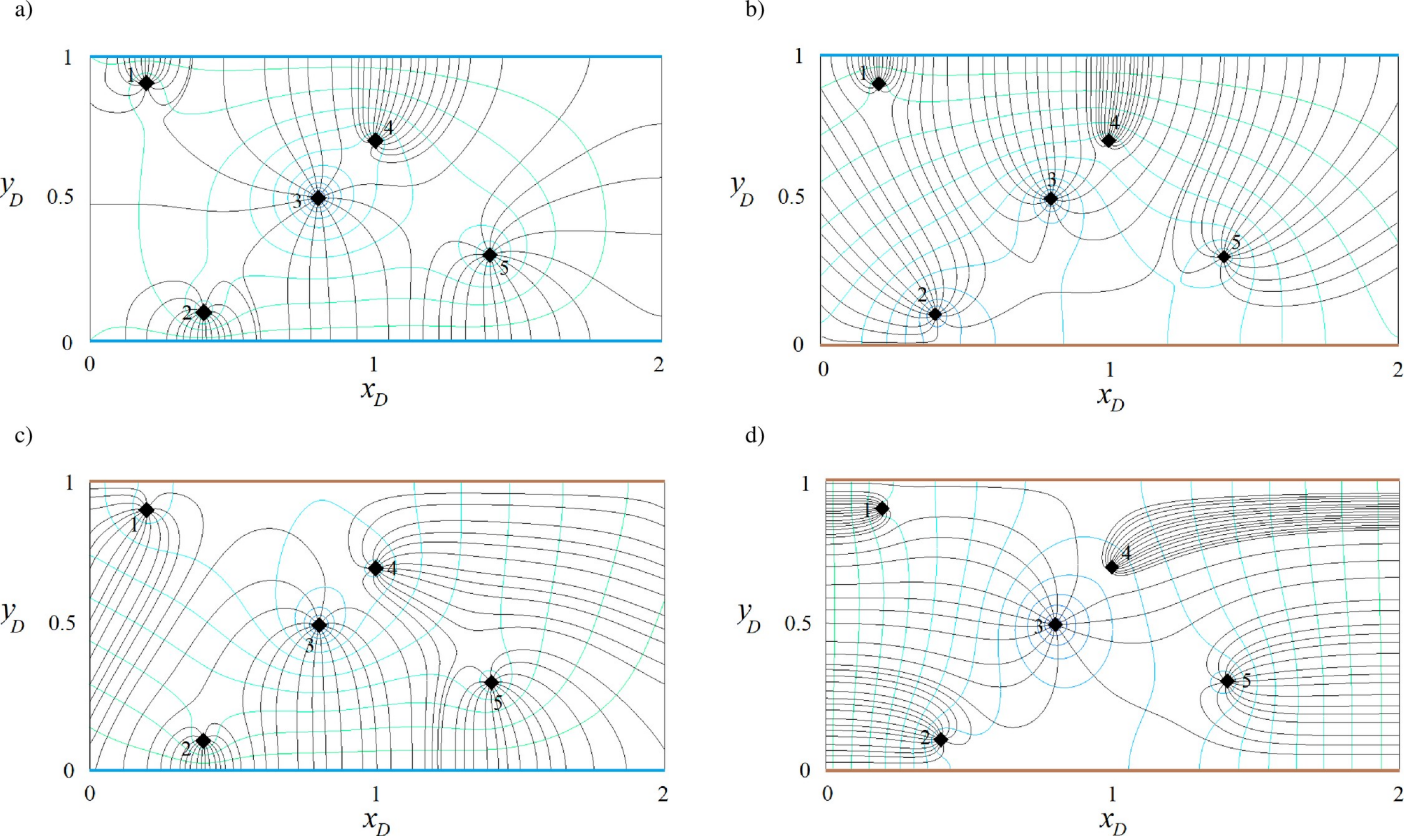
Capture zones of Fig 8 generated by numerical models.

To validate the developed capture zone solutions, the capture zones of 5 wells of Fig 8 were generated by MODFLOW 2000 [48] and MODPATH [49] and plotted in Fig 9. For the four plots shown in Fig 9, the well coordinates as well as their extraction/injection rates were set to those of Fig 8. The capture zones and the pattern of streamlines generated by the numerical models are comparable to the analytical model capture curves (i.e., Fig 8). The slight difference is mainly due to the approximation inherent in the numerical models. It is worth mentioning that the usage of the capture envelope equations developed in this paper is far easier, less time consuming, and more accurate than using the abovementioned numerical models, although numerical models are more flexible in dealing with complicated boundary conditions [10]. Figs 8 and 9 demonstrate the potential of developed analytical capture zone models to verify the accuracy of numerical models.

### Effects of the well distance from boundaries on the capture zones

**Fig 10:**
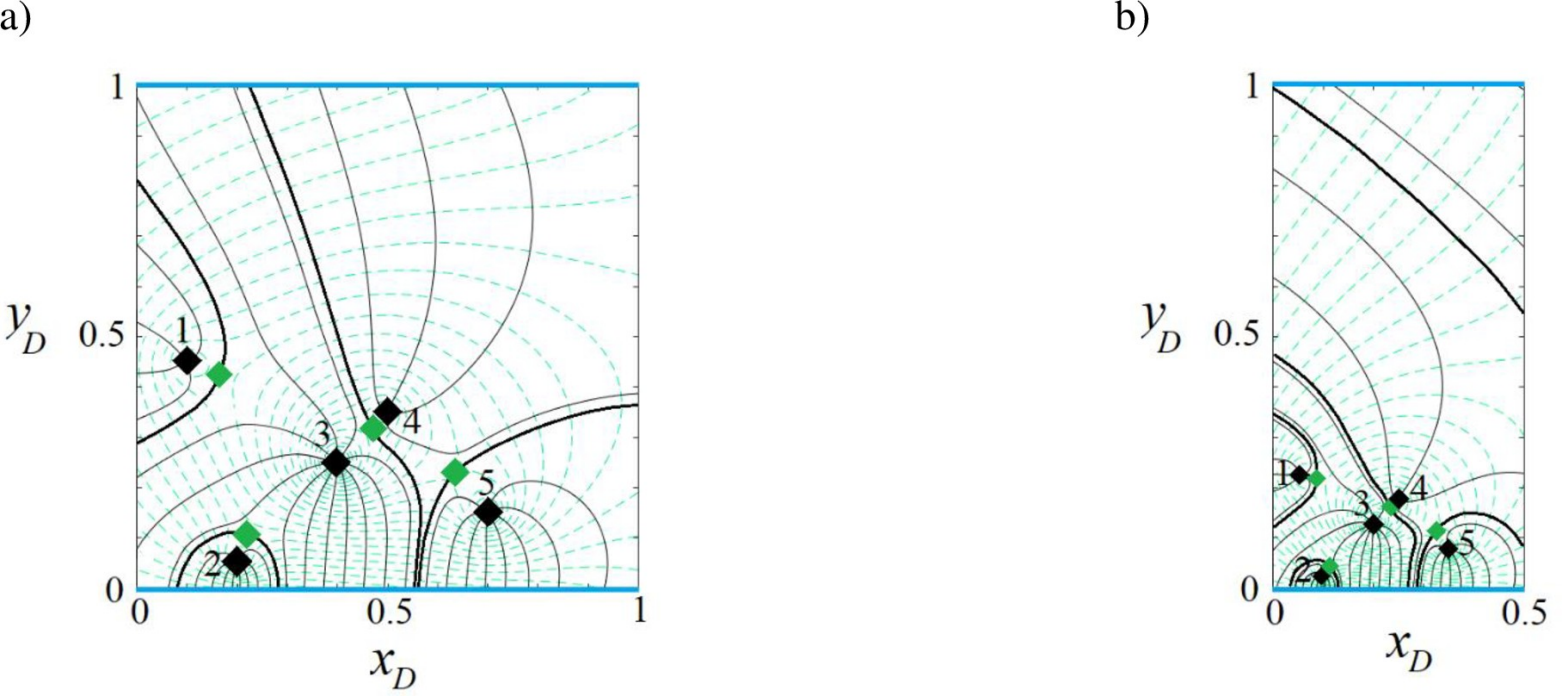
Velocity potential and streamlines when the well distances to the north boundary increased by (a) 2 and (b) 4 times, respectively compared to Fig 4D.

The positions of the wells are kept constant relative to the south side stream as in Fig 4D, and the northern boundary is moved away so that its distance to the wells increases by a factor of 2 (Fig 10A) or 4 (Fig 10B). Fig 10A illustrates that the capture zones are reshaped such that well (1) no longer is fed by the north side stream, but is instead fully fed by regional flow, while well (3) gains less water from the north side stream. Well (4), which was fully supplied with the north side stream, now gains water from both streams. Well (5), which was fed by both streams, is now recharged by the southern stream only. In Fig 10B, the distance between wells and the northern stream is increased, so that the capture zone of wells (1), (3) and (5) do not reach it, while well (1) is fed only by regional flow. Wells (3) and (5) are no longer supplied by the northern stream. The contribution of the north side stream to well (4) is also negligible. Practically speaking, for this case, the strip-shaped aquifer acts as an aquifer located within a semi-infinite spatial domain.

### Groundwater remediation scheme design

In this section, use of dimensionless capture type curves in water quality management such as groundwater remediation projects is demonstrated. A variety of remediation technologies can be used to ensure contaminant plumes are either contained, removed and/or treated so they no longer pose a threat to water resources. In a typical in situ remediation project such as bioremediation, pump-and-treat, plume containment, etc., polluted groundwater is extracted using one or more wells, treated and possibly reinjected [50]. In such projects the main elements of an efficient and cost-effective design are determination of the capture zone, the optimal number of wells, the optimal injection/extraction rates, and the layout of wells with respect to the plume [7–10, 18, 26, 50–52].

We consider a confined aquifer with the boundary configuration of type (a) in Fig 1. The problem setup includes known dimensions and hydraulic properties (aquifer thickness, length and width are 20 m, 1000 m and 500 m, respectively; *K* = 5 m/d and *q*_0_ = 0.1 m^2^/d). A contaminant plume is located along the direction of regional groundwater flow (from the west to the east, *β* = 0). The aim is to contain the plume hydraulically and prevent its extension downgradient using two or three wells. A logical step is to position an injection well (to inject treated water) in the vicinity of the contaminant source and to place one or more extraction wells at the leading edge of the plume. A solution could be determined manually by using Eqs (9) and (10) to generate a set of capture zone curves for various well layouts and extraction/injection rates. This approach, although cumbersome, could achieve a satisfactory plume capture design.

**Fig 11:**
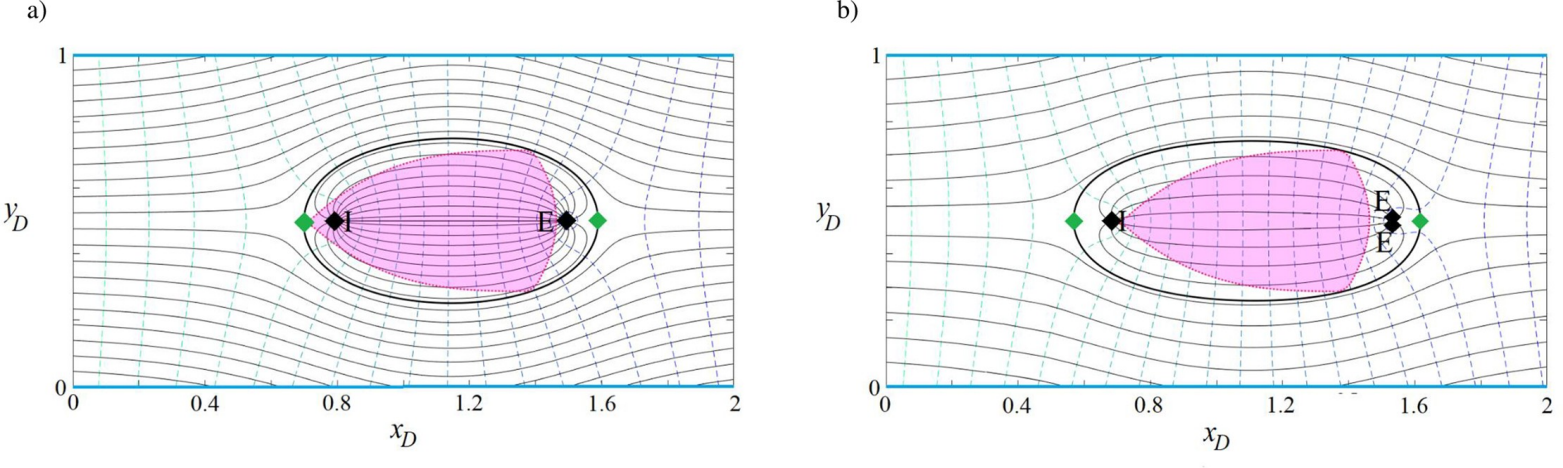
**Remediation of groundwater by the pump and treat method a) two-well and b) three-well system.** “I” shows the injection wells and “E” the extraction wells. The colored area is the original contaminant plume. The thick black line surrounding the plume is the dividing streamline that defines the capture zone that separates the plume from the rest of the aquifer and *β* = 0.

Two automated parameter optimization schemes are used instead, i.e., Eqs (9) and (10) are embedded into the PSO algorithm (Appendix B) and GA (Appendix C). Fig 10 illustrates the optimal capture envelopes for two- and three-well remediation schemes, with the corresponding well positions and extraction/injection rates given in Table 3. As is evident in Fig 11, both systems involve a closed region that contains and encompasses the plume. Table 3 shows that both optimization schemes result in similar optimal solutions. This approach can easily be extended to include financial constraints associated with well installation and operation. In Fig 11, the three-well solution has a lower total injection/extraction rate, so it represents the optimal solution if the remediation scheme is dominated by pumping costs. Fig 10 was also generated by the abovementioned numerical models and presented in Figure I in S1 File. In this simulation, if the well positions and flow rates were unknown, finding the optimum plan for the plume containment would be difficult to determine manually.

**Table 3:** Extraction/injection rates and well positions in two remediation scenarios.

Pump-and-treat remediation scenarios	Type of well	PSO			GA		
		*x*_*wD*_	*y*_*wD*_	*Q*_*wD*_	*x*_*wD*_	*y*_*wD*_	*Q*_*wD*_
**Two wells**	**Extraction**	1.4902	0.50	0.000736	1.5280	0.50	0.000796
	**Injection**	0.7976	0.50	0.000736	0.6759	0.50	0.000796
**Three wells**	**Extraction 1**	1.5314	0.4932	0.000288	1.5579	0.4957	0.000306
	**Extraction 2**	1.5314	0.5068	0.000288	1.5579	0.5043	0.000306
	**Injection**	0.6845	0.5	0.000576	0.6357	0.5	0.000612

Dimensional values of parameters (i.e., *x*_*w*_, *y*_*w*_, *Q*_*w*_) can be calculated using Eq (7).

### Water quantity management projects

**Fig 12:**
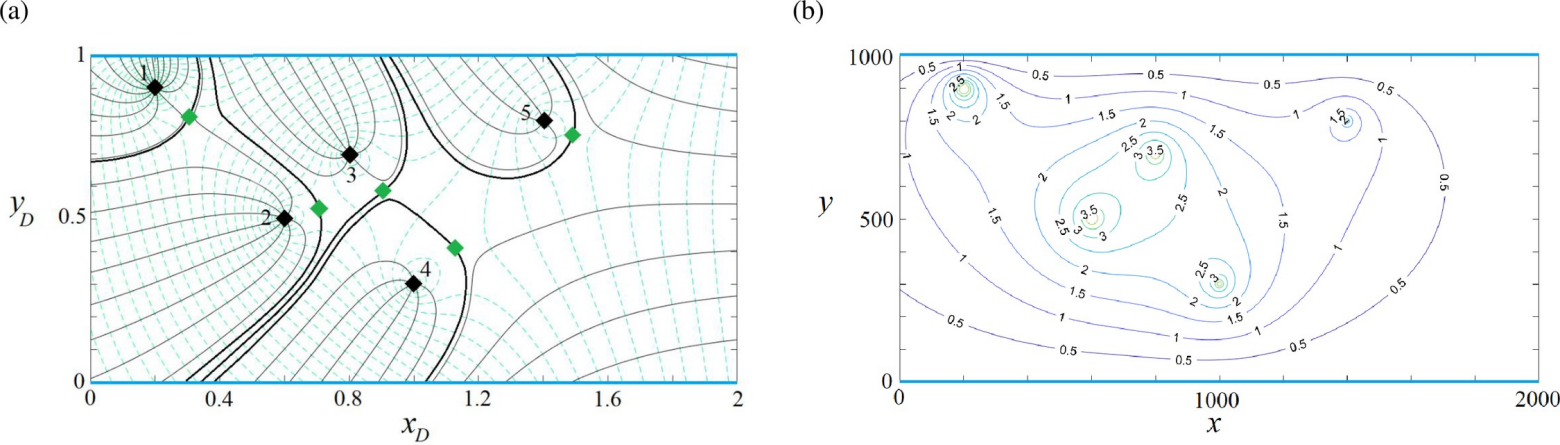
a) Velocity potential and streamlines of a water quantity management project in a strip-shaped aquifer with inflow-inflow boundary conditions, *β* = 0. b) Corresponding dimensional drawdown contours (m).

The availability and sustainability of groundwater in many major aquifers in the world are threatened due to excessive depletion. To avoid this, a typical management strategy is to allocate well pumping rates in such a way that the drawdown in the aquifer does not exceed a certain value (permissible drawdown). As an example, we consider a confined aquifer with the boundary configuration of type (a) in Fig 1. The problem setup includes known dimensions and hydraulic properties (aquifer thickness, length and width are 20 m, 1000 m and 500 m, respectively; *K* = 5 m/d and *q*_0_ = 0.1 m^2^/d). Further, it is assumed that the aquifer is pumped by five wells with positions given in Table 4. The objective of this example is to calculate extraction rates such that the maximum drawdown is less than 4 m (Fig 12).

**Table 4:** Well positions and the calculated extraction rates of wells in the quantity management scenario.

*Well number*	Well coordinates				PSO		GA	
	*x*_*wD*_	*y*_*wD*_	*x*_*w*_(*m*)	*y*_*w*_(*m*)	*Q*_*wD*_	*Q*_*w*_(*m*^3^/*d*)	*Q*_*wD*_	*Q*_*w*_(*m*^3^/*d*)
**1**	0.2	0.9	100	450	0.01	500	0.0093	465
**2**	0.6	0.5	300	250	0.0058	290	0.0057	285
**3**	0.8	0.7	400	350	0.005	250	0.0055	275
**4**	1	0.3	500	150	0.0044	220	0.0045	225
**5**	1.4	0.8	700	400	0.0032	160	0.0034	170

Based on Eq (7), the wells and the aquifer parameter values are converted to dimensionless values and Eq (15) is solved using the abovementioned optimization algorithms. The calculated optimum pumping rates by both optimization algorithms are presented in Table 4 with similar results. The capture envelope patterns of the 5 wells for the optimum solution are presented in Fig 11A and the corresponding dimensional drawdown contours calculated by Eq (15) are shown in Fig 11B. Note that the permissible 4-m drawdown is not exceeded. Table 5 presents the definition of variables in the equations.

**Table 5:** Mathematical Notations.

*b*	Aquifer thickness	L
*c*	+1 or –1 for extraction and injection wells, respectively	
*c*_1_, *c*_2_	Acceleration constants in the PSO algorithm	
*d*	Distance between two boundaries	L
*d*_*D*_	Dimensionless distance between two boundaries	
*j*	Summation index	
*h*	Hydraulic head	L
*h*_*D*_	Dimensionless hydraulic head	
*h*_*w*_	Hydraulic head at *r*_*w*_	L
*h*_*wD*_	Dimensionless hydraulic head at *r*_*w*_	
${\bar{h}}_{0}$	Average of the initial hydraulic head	L
*K*	Hydraulic conductivity	LT^-1^
*l*_*Dp*_	Dimensionless plume length	
*N*	Number of wells	
*q*_0_	Regional uniform flow per unit width	L^2^T^-1^
*q*_0*D*_	Dimensionless regional uniform flow per unit width	
*Q*_*w*_	Pumping or injection rate	L^3^T^-1^
*Q*_*wD*_	Dimensionless pumping or injection rate	
*Q*_*wDj*_	Dimensionless pumping or injection rate of the *j*^th^ well	
*r*	Distance from well	L
*r*_*w*_	Well radius	L
*r*_1_, *r*_2_	Random real numbers between 0 and 1 in the PSO algorithm	
*s*	Drawdown	L
*s*_*D*_	Dimensionless drawdown	
*s*_*p*_	Permissible drawdown	L
*v*	Velocity	LT^-1^
*w*_*Dp*_	Dimensionless plume width	
*x*_*Dc*_	Dimensionless capture zone length	
*x*_*wDj*_	Dimensionless *x* position of the *j*^th^ well	
*y*_*Dc*_	Dimensionless capture zone width	
*y*_*wDj*_	Dimensionless *y* position of the *j*^th^ well	
*z*_1_	Complex coordinate in the physical plane	
*z*_2_	Complex coordinate in the *z*_2_−*plane*	
*z*_3_	Complex coordinate in the *z*_3_−*plane*	
*z*_*wj*_	Coordinate of the *j*^th^ extraction or injection well	
*z*_*wDj*_	Dimensionless coordinate of the *j*^th^ extraction or injection well	
*Greek*		
*β*	Angle of flow direction with the *x* axis	rad
*δ*	Coordinate of the extraction or injection well in the *z*_3_−*plane*	
$\bar{\delta }$	Complex conjugate of *δ*	
*δ*_*j*_	Coordinate of the *j*^th^ extraction or injection well in the *z*_3_−*plane*	
*ζ*	Coordinate of the extraction or injection well in the *z*_2_−*plane*	
*ϕ*	Discharge potential (real part of the complex potential function, *Ω*)	L^3^T^-1^
*ϕ*_0_	Initial potential along the inflow boundary	L^3^T^-1^
*ϕ*_*D*_	Dimensionless *ϕ*	
*ϕ*_*w*_	Potential at well	L^3^T^-1^
*ϕ*_*wD*_	Dimensionless potential at well	
*χ*	Constriction coefficient in the PSO algorithm	
*ψ*	Stream function (imaginary part of complex potential function, *Ω*)	L^3^T^-1^
*ψ*_*D*_	Dimensionless *ψ*	
*Ω*	Complex potential function	L^3^T^-1^
*Ω*_*D*_	Dimensionless complex potential function	
*ω*	Inertia weight in the PSO algorithm	

## Conclusions

Analytical models of the capture zone (stream function, velocity potential and stagnation point) for a multi-well system with any number of extraction/injection wells in confined and unconfined strip-shaped aquifers, with/without uniform regional flow are developed from the theory of complex velocity potentials and the Schwarz-Christoffel conformal mapping. The solution is provided for four strip boundary configurations: barrier-barrier, barrier-inflow, inflow-barrier and inflow-inflow. The solution, which is straightforward to compute, can be used to generate dimensionless capture type curves. The applicability of the developed model to evaluate surface-subsurface water interaction, well-head protection plans and water rights adjudication–which are key factors in the management of water resources–were demonstrated through numerical examples (Figs 4–8 and 12). These examples also displayed that the size and pattern of the capture envelopes are controlled by the extraction/injection rate, distance of wells from the boundaries, distances between wells, type and number of wells, the rate and direction of regional flow and the initial head along the boundaries. Further, the derived solution was embedded with two optimization schemes (PSO and GA) for the determination of optimal plume capture schemes for pump-and-treat remediation projects and for determination of the optimal pumping policy for sustainable water extraction schemes, with similar results obtained by both methods. The solution was validated by numerical models, and is thus suitable for quantifying the accuracy of such models for complex water injection/extraction scenarios. In general, the proposed models can be used by hydrogeologists, engineers and planners to design optimal layouts of groundwater remediation projects (e.g., in situ bioremediation or chemical oxidation schemes) that involve pump-and-treat and plume containment operations, to design the most cost-effective pumping schemes for sustainable development of water resources, to study the interaction of surface and groundwater resources and to verify numerical models.

**Appendix A**

$$f_{1Dj}=\exp\left(\frac{\pi x_{D}}{2}\right)\cos\left(\frac{\pi y_{D}}{2}\right)-\exp\left(\frac{\pi x_{wDj}}{2}\right)\cos\left(\frac{\pi y_{wDj}}{2}\right)$$

$$f_{2Dj}=\exp\left(\frac{\pi x_{D}}{2}\right)\sin\left(\frac{\pi y_{D}}{2}\right)-\exp\left(\frac{\pi x_{wDj}}{2}\right)\sin\left(\frac{\pi y_{wDj}}{2}\right)$$

$$g_{1Dj}=\exp\left(\frac{\pi x_{D}}{2}\right)\cos\left(\frac{\pi y_{D}}{2}\right)+\exp\left(\frac{\pi x_{wDj}}{2}\right)\cos\left(\frac{\pi y_{wDj}}{2}\right)$$ (A-1)

$$g_{2Dj}=\exp\left(\frac{\pi x_{D}}{2}\right)\sin\left(\frac{\pi y_{D}}{2}\right)+\exp\left(\frac{\pi x_{wDj}}{2}\right)\sin\left(\frac{\pi y_{wDj}}{2}\right)$$

**Appendix B. Particle Swarm Optimization (PSO)**

The PSO algorithm mimics birds searching for food. Two factors are considered, an individual’s previous best experience (*pbest*) and the best experience of all other individuals (*gbest*). The PSO method follows these steps [45]:

1. Define the objective (fitness) function and constraints. The objective of the pump-and-treat remediation model is to optimize extraction/injection rates to contain the contaminant plume by minimizing the objective function (*F*) under given constraints. The objective function and constraints are:
  $$Minimize\;F=\frac{{\left(y_{Dc}-w_{Dp}\right)}^{2}}{{w_{Dp}}^{2}}+\frac{{\left(x_{Dc}-l_{Dp}\right)}^{2}}{{l_{Dp}}^{2}}$$ (B-1)
  subject to:
  $$Q_{jmin}\leq Q_{wjD}\leq Q_{jmax}$$
  $$x_{jmin}\leq x_{wjD}\leq x_{jmax}$$ (B-2)
  $$y_{jmin}\leq y_{wjD}\leq y_{jmax}$$
  $$s_{wjD}\leq s_{jmax}$$
  where *y*_*Dc*_ is the *y*-coordinate of capture envelope based on Eq (9), *w*_*Dp*_ is plume width, and *x*_*Dc*_ and *l*_*Dp*_ are the length of the capture envelope and plume, respectively. Also, *Q*_*jmin*_ and *Q*_*jmax*_ are minimum and maximum injection/extraction rates for the *j*^*th*^ well. The locations *x*_*jmin*_, *x*_*jmax*_, *y*_*jmin*_ and *y*_*jmax*_ are problem-specific and are defined by user. The maximum permissible drawdown in the extraction wells is *s*_*jmax*_.
2. Create a population of particles–In the context of the pump-and-treat problem these are the initial extraction/injection rates (*Q*_*w*_) and well coordinates (*z*_*w*_), assigned between the lower and upper bounds of the constraints (Eq B-2). With these initial values, the capture zone is determined by Eqs (9) and (10). Each particle (well) also has an injection/extraction rate, which is used to find the new particle position based on Eqs (B-3) to (B-6) below.
3. Calculate the fitness value (Eq B-1). The fitness value is accepted if the capture envelope contains the contamination plume. Otherwise, *z*_*w*_ and *Q*_*w*_ are changed by considering the best estimated injection/extraction rate of all wells (*gbest*) that has the minimum fitness value. Based on Eqs (B-1) and (B-2) and the best estimated injection/extraction rate of each well (*pbest*), the iteration continues. In the first iteration, the initial value of well rates and coordinates are the *pbest* values.
4. Compute the new rates and well coordinates of the particles (wells) according to:
  $$v_{i}^{t}=\chi \left[\omega v_{i}^{t-1}+c_{1}r_{1}(z_{wpi}^{t-1}-z_{i}^{t-1})+c_{2}r_{2}(z_{wg}^{t-1}-z_{i}^{t-1})\right]$$ (B-3)
  $$z_{wi}^{t}=z_{wi}^{t-1}+v_{i}^{t}$$ (B-4)
  $$v_{i}^{t}=\chi \left[\omega v_{i}^{t-1}+c_{1}r_{1}(Q_{wpi}^{t-1}-z_{i}^{t-1})+c_{2}r_{2}(Q_{wg}^{t-1}-z_{i}^{t-1})\right]$$ (B-5)
  $$Q_{wi}^{t}=Q_{wi}^{t-1}+v_{i}^{t}$$ (B-6)
  where *c*_1_ and *c*_2_ are acceleration constants (learning factors), *r*_1_ and *r*_2_ are random numbers uniformly distributed between 0 and 1, subscripts *p* and *g* respectively indicate the *pbest* and *gbest* values of the particles, *ω* is the inertia weight used to control the impact of the previous history of velocities on the current value, *χ* is the constriction coefficient, applied to restrain the velocity (*v*), number of iterations (*t*), well rates (*Q*_*w*_) and the particle position (*z*_*w*_).
5. Repeat steps 3 and 4 until the fitness criterion or the maximum number of iterations is reached.

The above procedure was performed within MATLAB.

For water quantity management projects, the aim is to optimize extraction rates contingent upon a certain value of drawdown (permissible drawdown, *s*_*p*_) where the objective function and constraints are:

$$Maximize\;Q_{wjD}$$ (B-7)

subject to:

$$Q_{jmin}\leq Q_{wjD}\leq Q_{jmax}$$

$$s_{max}\leq s_{p}$$ (B-8)

where, *Q*_*wjD*_ is the extraction rates based on Eq (8), *Q*_*jmin*_ and *Q*_*jmax*_ are user-defined minimum and maximum extraction rates for the *j*^*th*^ well.

**Appendix c. genetic algorithm**

The concept of genetic algorithm (GA) to optimization was first introduced by Holland [53]. The concept inspired by the process of natural selection consisted of selection, crossover, mutation, reproduction and replacement searches for a global optimal or a near‐global optimal solution. For a comprehensive understanding of GA and its application in water resources management, readers are referred to [54–59]. The GA method follows these steps:

1. Define objective function (fitness) and constraints. The objective of the pump-and-treat remediation model is to optimize extraction/injection rates to contain the contaminant plume by minimizing the objective function (Eq B-1 or B-7) under given constraints (Eq B-2 or B-8).
2. Assign an initial population for the parameters to be optimized (position and rate of wells).
3. Create a sequence of new populations. At each step, the algorithm uses the individuals in the current generation to create the next population. To create the new population, the algorithm performs the following steps:
  - Score the current population by computing its fitness value. These values are called the raw fitness scores.
  - Scale the raw fitness scores to convert them into a more usable range of values. These scaled values are called expectation values.
  - Some of the individuals in the current population that have lower fitness are selected as *elite*. These elite individuals are passed to the next population.
  - Produce children from the parents. Children are produced either by making random changes to a single parent (*mutation*) or by combining the vector entries of a pair of parents (*crossover*).
  - Replace the current population with the children to form the next generation.

The above steps are repeated until some criteria such as the number of generations (iterations), time limit or fitness limit are achieved.

## Supporting information

S1 File Mapping the conceptual model from the physical plane *z*_1_ to the imaginary planes *z*_2_ and z_3_, and effect of regional flow rate and direction on the shape and orientation of well capture zones. (DOCX)
