# Supplementary material for: Multi-well capture zones in strip-shaped aquifers
Source: PLoS One. 2020 Mar 5;15(3):e0229767. doi: 10.1371/journal.pone.0229767 (PMC7058347; doi:10.1371/journal.pone.0229767)
Supplement: S1 File — (DOCX) [file pone.0229767.s001.docx]

**Supporting information**

Multi-well capture zones in strip-shaped aquifers

Setareh Nagheli^1,2^, Nozar Samani^1*^, D. A. Barry^2^

^1^*Department of Earth Sciences, Shiraz University, Shiraz, 7146713565, Iran*

^2^*Laboratoire de technologie écologique (ECOL), Institut des sciences et technologies de l’environnement (IIE), Faculté de l’environnement naturel, architectural et construit (ENAC), Ecole polytechnique fédérale de Lausanne (EPFL), Lausanne, Switzerland*

*^*^Corresponding author:*

*Email:* [*samanin@shirazu.ac.ir*](mailto:samanin@shirazu.ac.ir)

**Mapping the conceptual model from the physical plane *z*_1_ to the imaginary planes *z*_2_ and *z*_3_**

In the Schwarz-Christoffel conformal mapping, we consider the mapping $z_{1}$ *= F* ($z_{2}$) for which [44]:

$\frac{dz_{1}}{dz_{2}}=C{(z_{2}-\xi_{1})}^{k_{1}}{(z_{2}-\xi_{2})}^{k_{2}}{(z_{2}-\xi_{3})}^{k_{3}}{(z_{2}-\xi_{4})}^{k_{4}}$ (S1)

where $\xi_{1}$, $\xi_{2}$,$\xi_{3}$, $\xi_{4}$ are any *n* points arranged consecutively along the real axis in the $z_{2}-plane$ (Fig S1), *C* is a real or complex constant and $k_{n}=1-\alpha_{n}/\pi$ where $\alpha_{n}$ is the interior angle of the resultant polygonal contour at the point *z_n_*. Integrating Eq (S1) results in:

$z_{1}=C_{1}\int\prod_{n=1}^{4} {(z_{2}-\xi_{n})}^{-k_{n}}dz_{2}+C_{2}$ (S2)

where, *C*_1_ and *C*_2_ are complex constants calculated using the boundary conditions [44]. The origin in the $z_{1}$and $z_{2}-plane$ is chosen as shown in Figure A, with the *x*-axis coinciding with one of the boundaries. The aquifer is transformed to the upper half $z_{2}-plane$ and points 1 and 3 are chosen at infinity and at the origin of the $z_{2}-plane$, respectively. After transformation, the coordinates of points 1 to 4 in the $z_{2}-plane$ are:$\xi_{1A}=-\infty$, $\xi_{2}=-1$, $\xi_{3}=0$, $\xi_{4}=+1$ and $\xi_{1B}=+\infty$, $k_{1}$ = $k_{3}$ = $1$ and $k_{2}$ = $k_{4}$ = 0. By substituting the boundary conditions into Eq (S2), one can derive.

$z_{1}=C_{1}\int\frac{dz_{2}}{z_{2}}+C_{2}$ (S3)

Substituting the position of points 4 and 2 in the $z_{1}$ and $z_{2}-planes$ into Eq (S3) results in:

$z_{1}=\frac{d}{\pi}\ln\left( z_{2} \right)$ (S4)

given in Capture zone Section as$z_{2}=\exp\left( {\pi z_{1}}/d \right).$


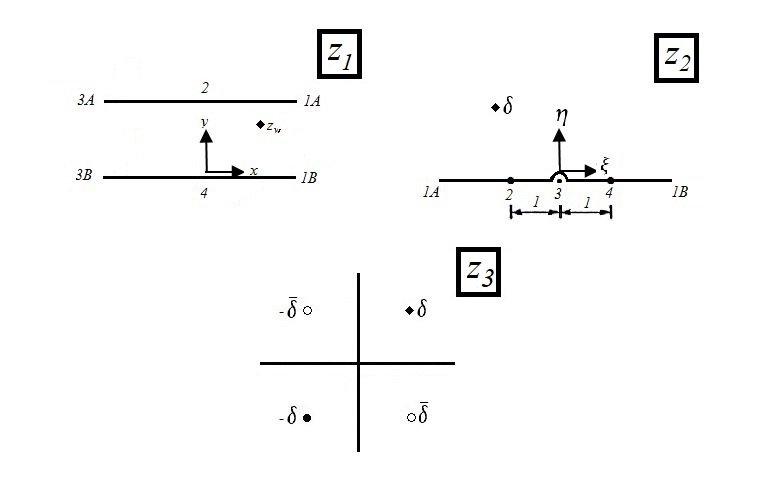


**Figure A. Mapping the conceptual model (Fig1a) from the physical plane** $\boldsymbol{z}_{\boldsymbol{1}}$ **to the** $\boldsymbol{z}_{\boldsymbol{2}}$ **and** $\boldsymbol{z}_{\boldsymbol{3}}$**.**

When the boundaries are a combination of barrier-inflow and inflow-barrier, the $z_{2}-plane$ is transformed into the the $z_{3}-plane$, using the transformation $z_{3}={z_{2}}^{1/2}$. Writing Eq (S5) for the $z_{3}-plane$ results in $z_{1}={2d\ln\left( z_{3} \right)}/\pi$ which is expressed in Eq (1) as $z_{3}=exp\left( {\pi z_{1}}/{2d} \right)$.

**Branch cut**

| 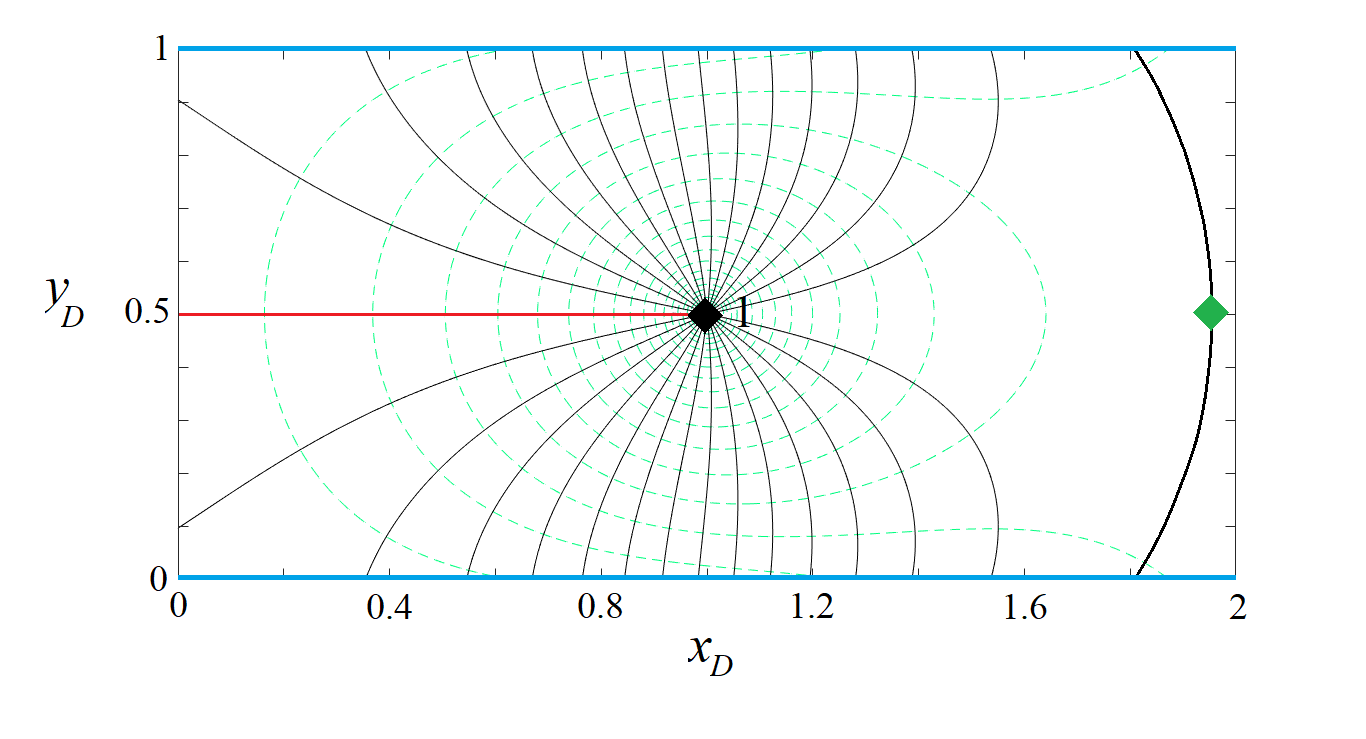 | 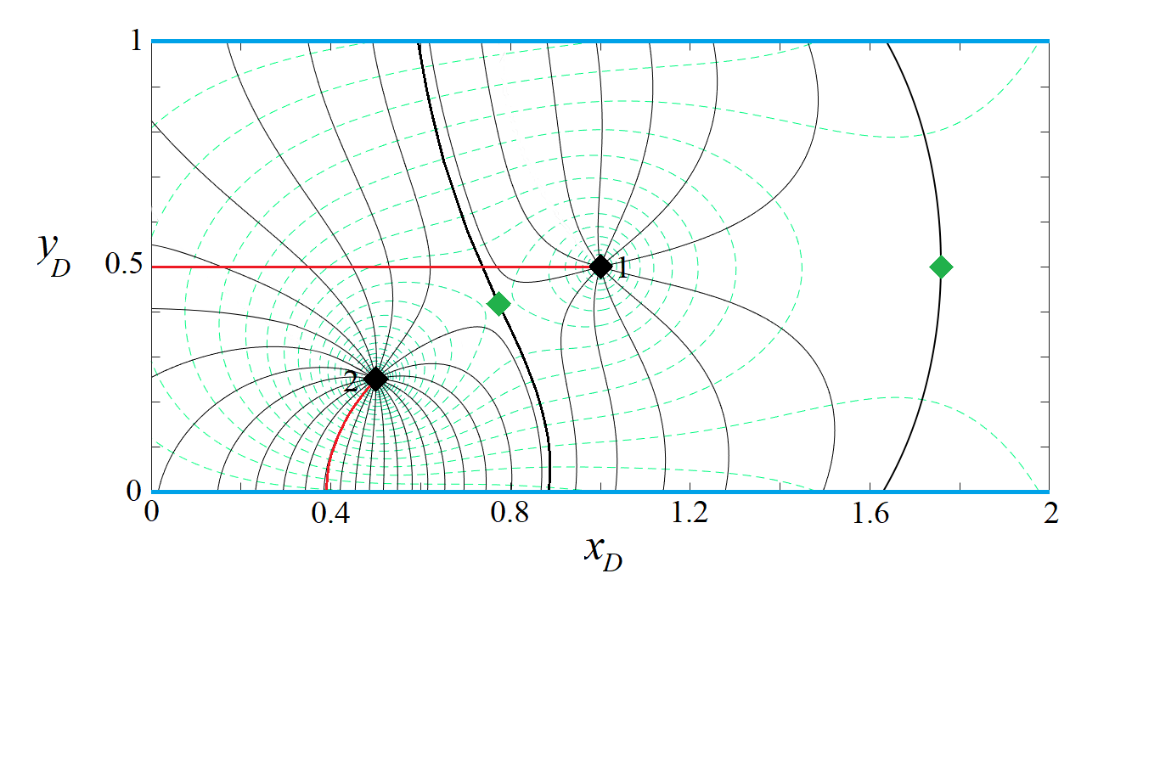 |
| --- | --- |
| c) | d) |
| 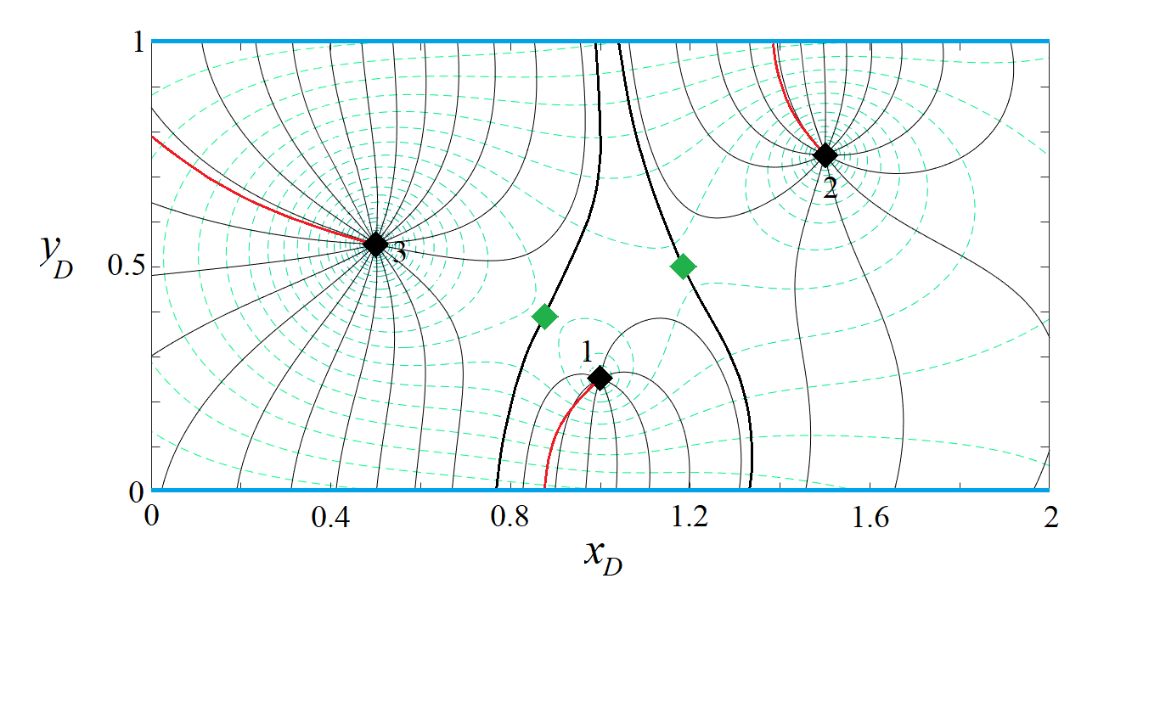 | 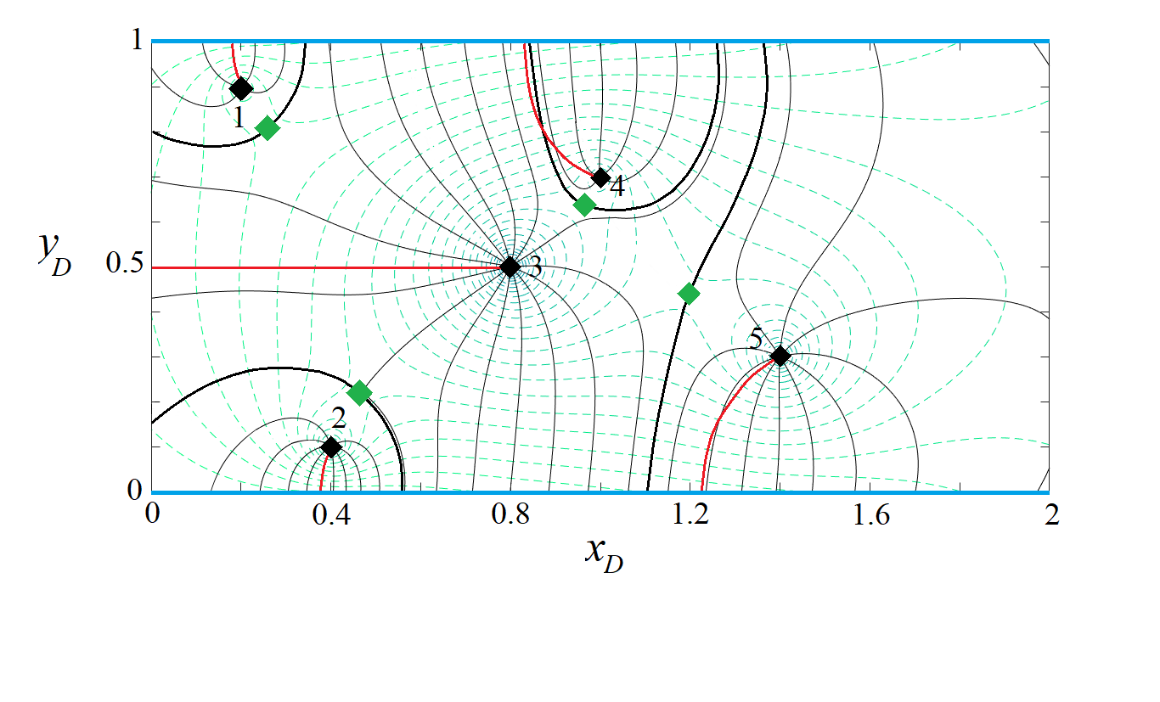 |
|  | |

**Figure B. Fig 4 with branch cuts (red lines).**

**Effect of regional flow rate and direction on the shape and orientation of well capture zones**

Figure C is plotted for $\beta=\pi/2$ corresponding to Figs 4b and 4d. Figs D-H are plotted for the same conditions as Figs 4-8 except for the regional dimensionless flow rate $(q_{0D})$, which is equal to 0.02. These figures demonstrate the profound effect of regional flow rate and direction on the size, shape, dimension and pattern of well capture zones and the interaction of aquifer-well-boundary systems.

| a) | b) |
| --- | --- |
| 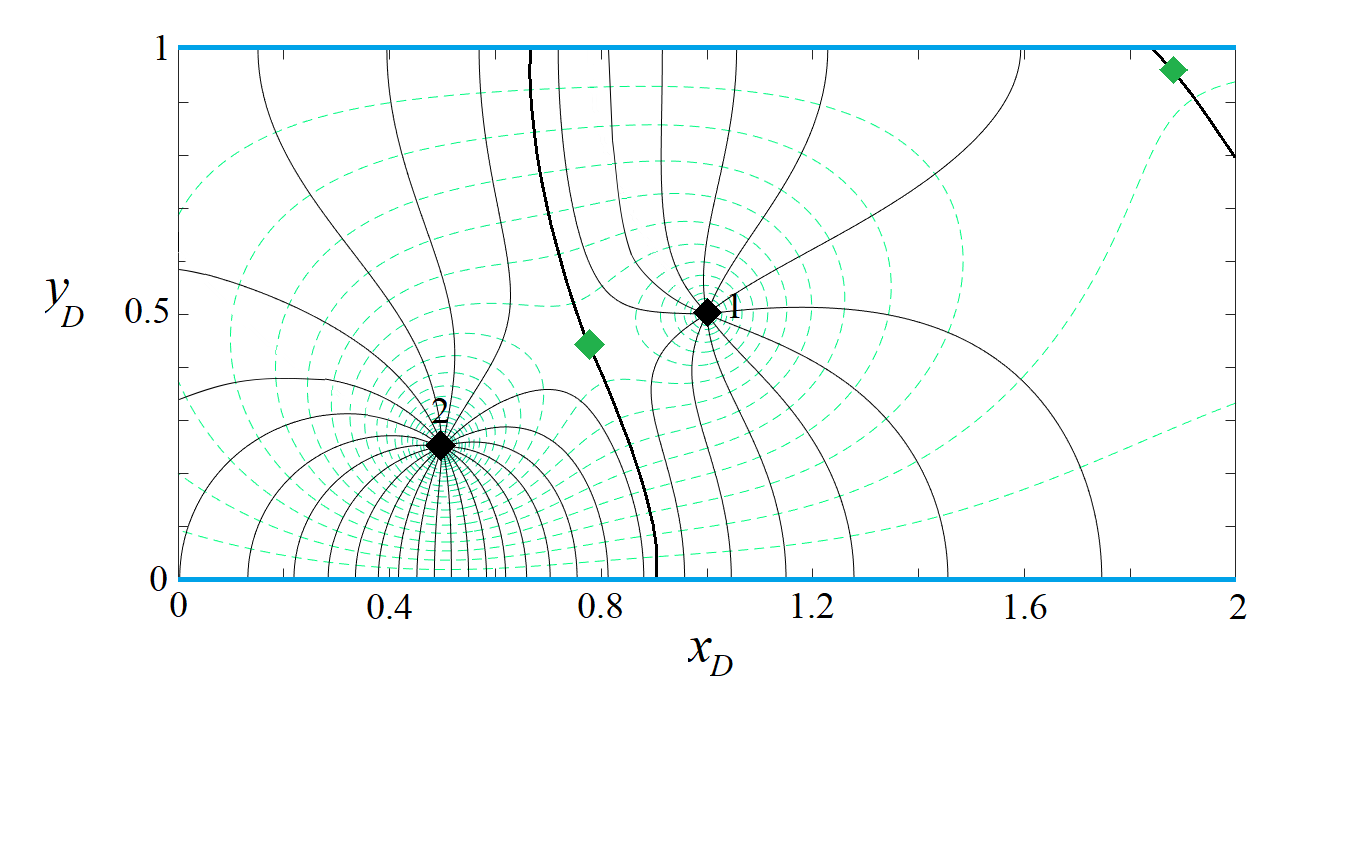 | 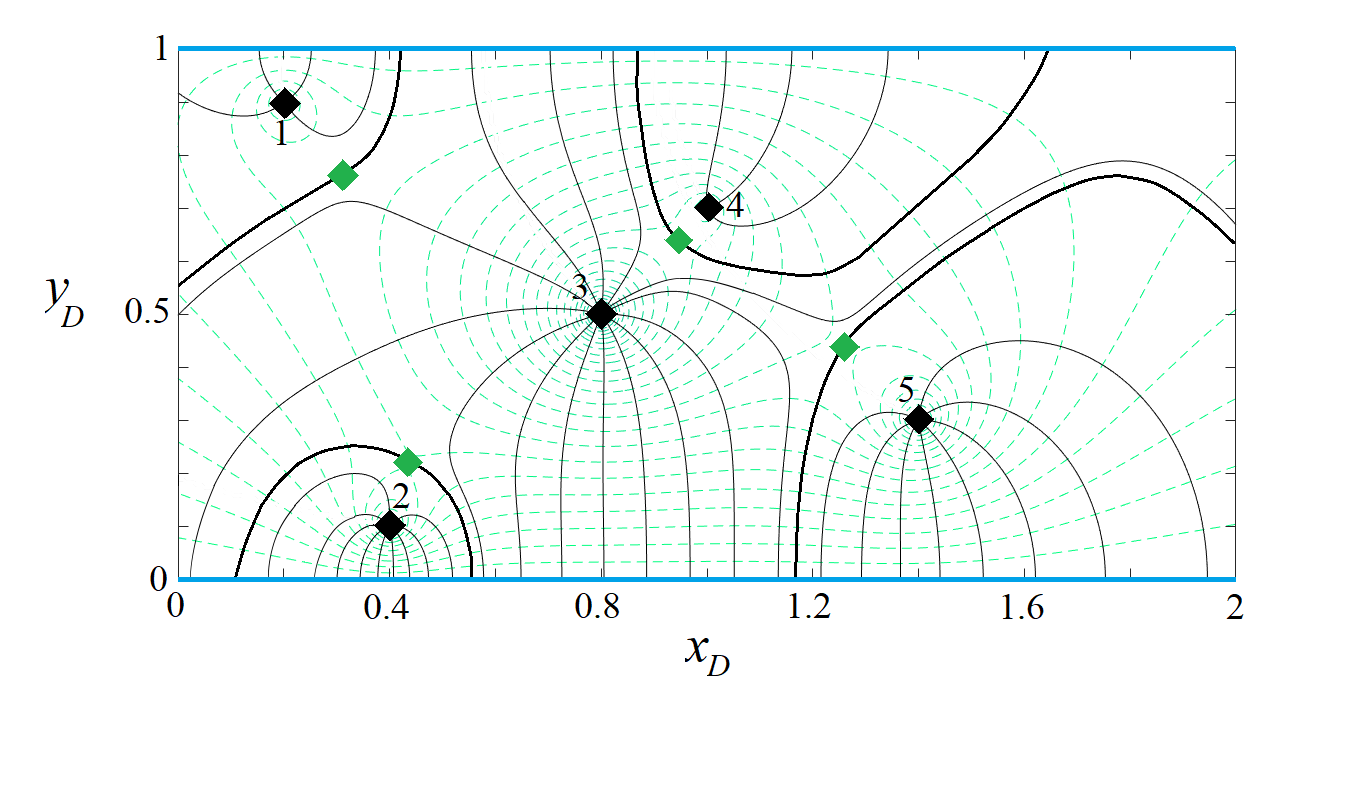 |
| **Figure C. Velocity potential and streamlines when** $\boldsymbol{\beta=}\boldsymbol{\pi}/\boldsymbol{2}$ **in a strip-shaped aquifer with inflow-inflow boundary conditions as in Figs 4b and d.** | |

| a) | b) |
| --- | --- |
| 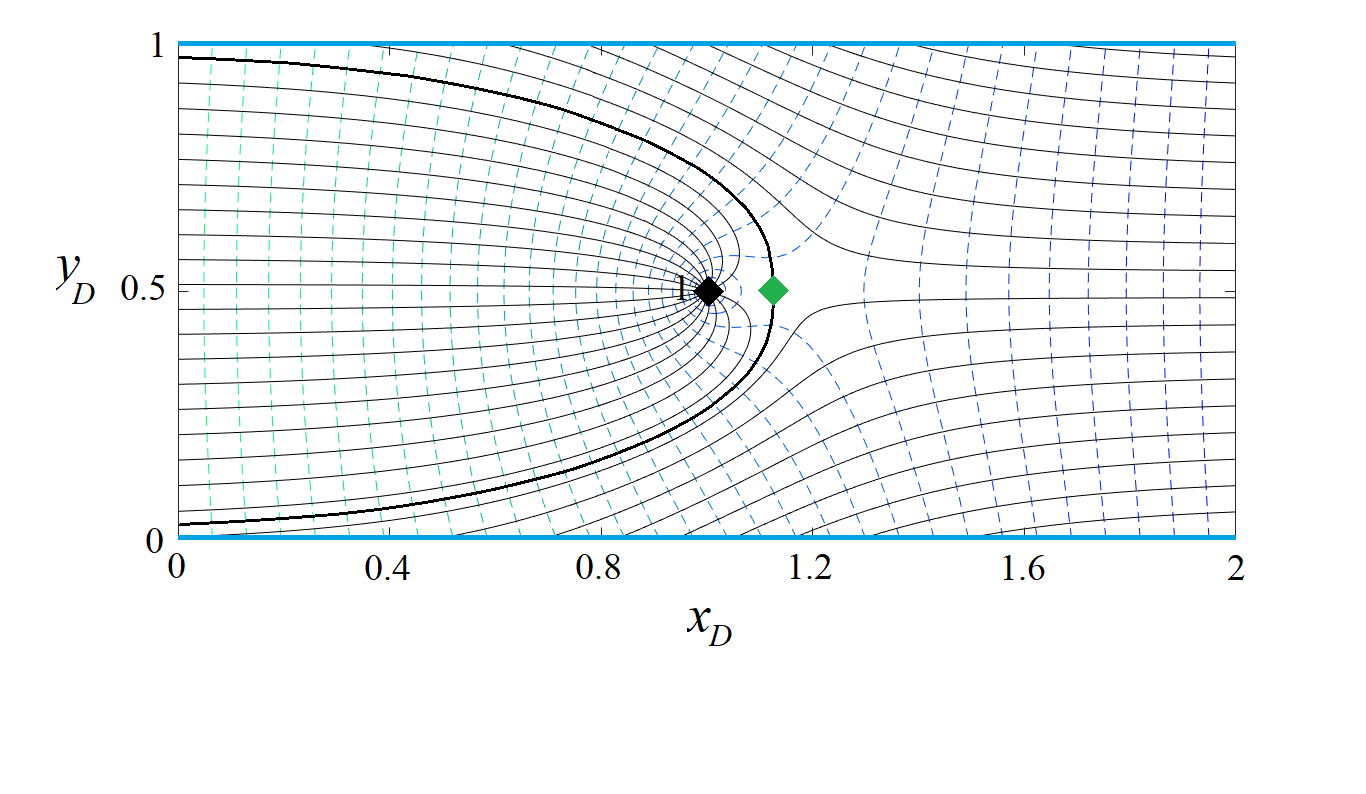 | 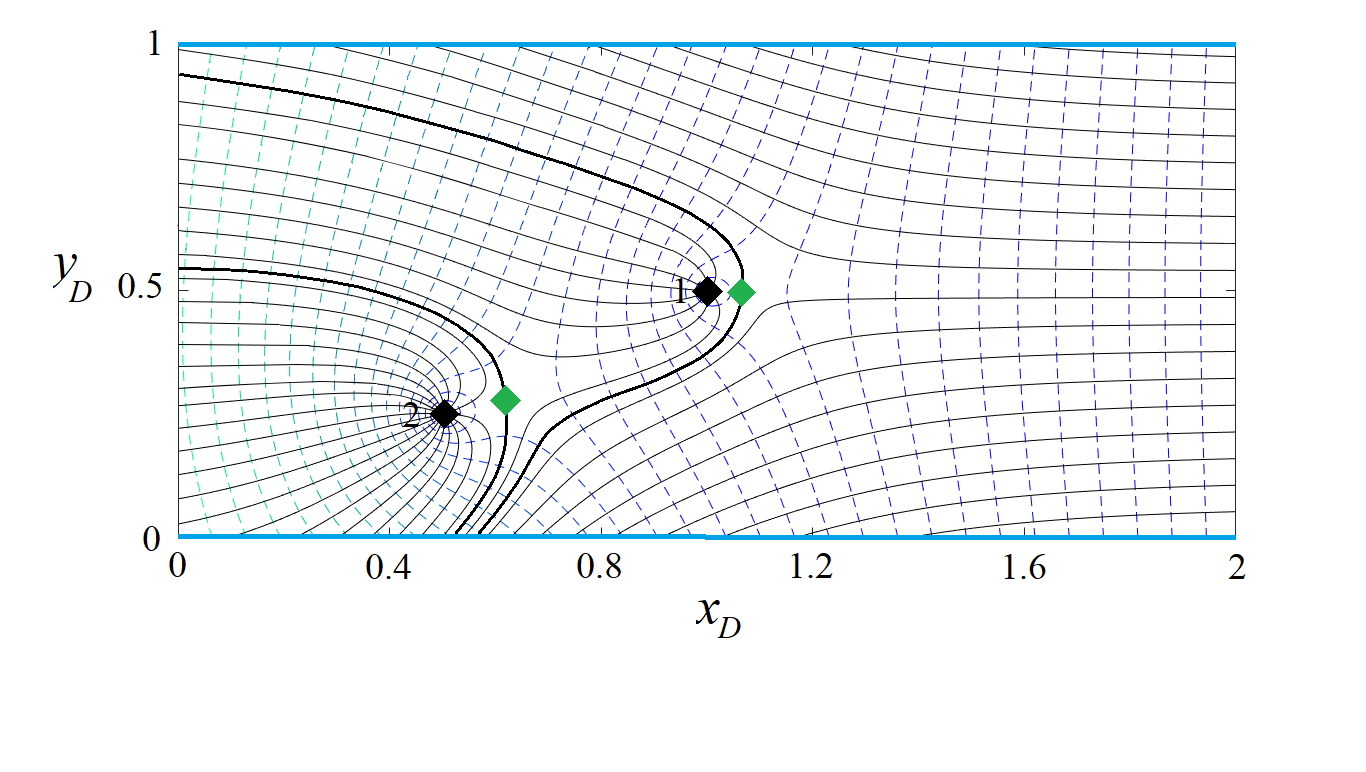 |
| c) | d) |
| 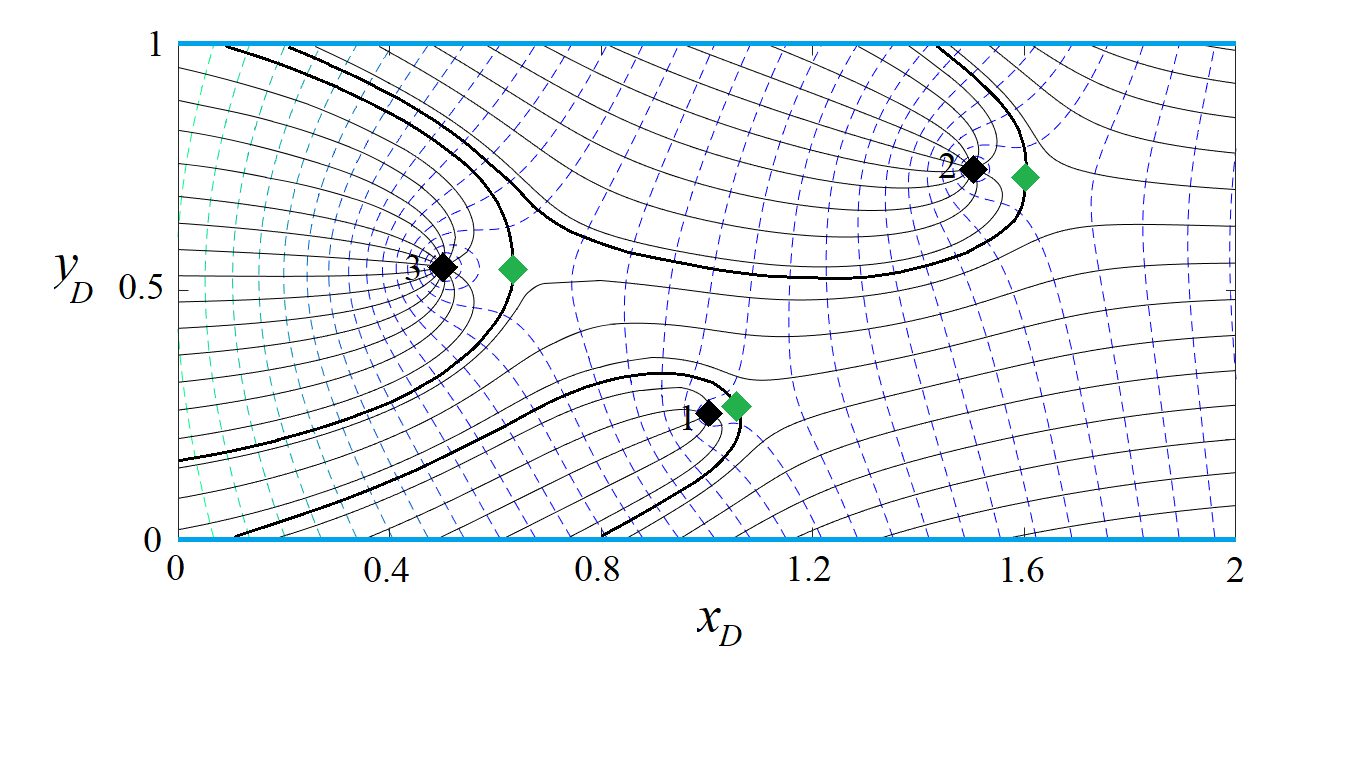 | 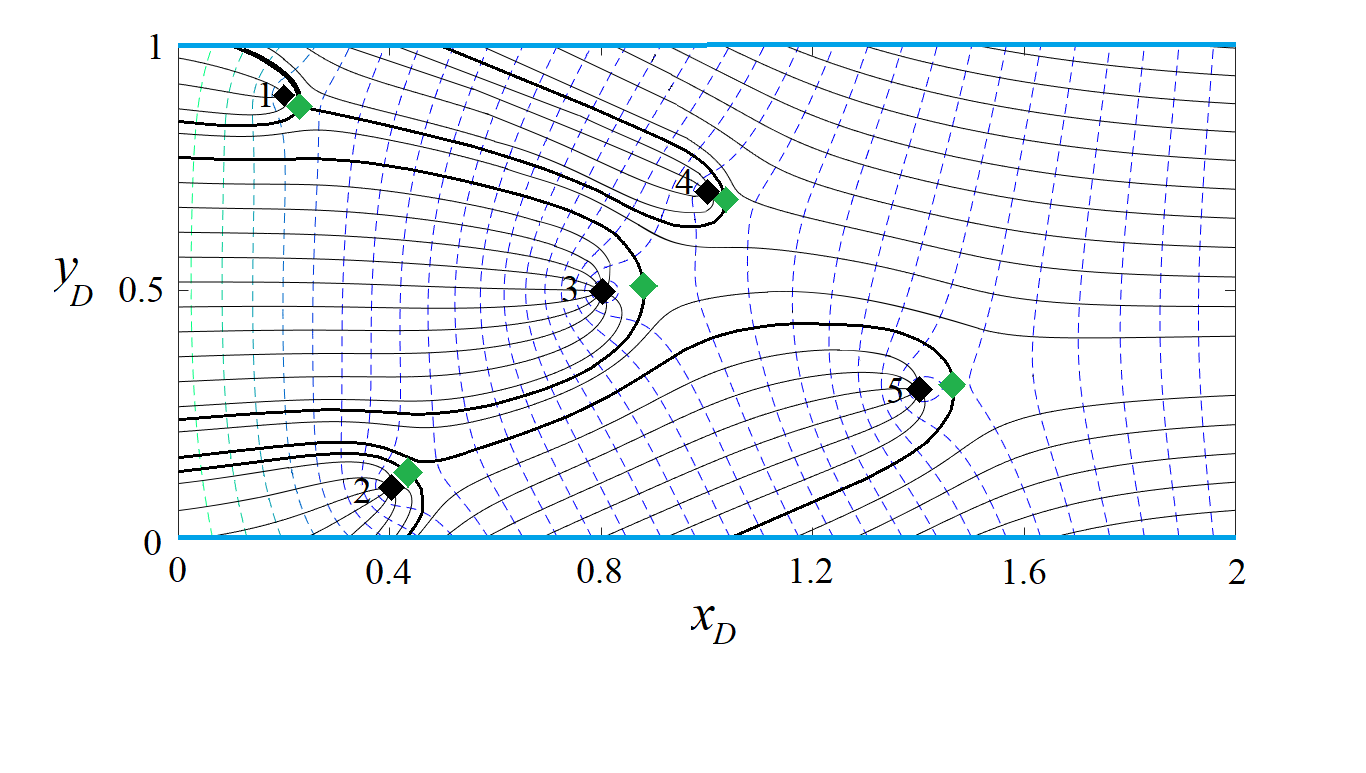 |
| **Figure D. Velocity potential (dashed lines) and stream function (solid lines) for one (a), two (b), three (c) and five (d) wells in a strip-shaped aquifer with inflow-inflow boundary conditions for** $\boldsymbol{\beta}\mathbf{=0}$ **and** $\boldsymbol{q}_{\boldsymbol{0}\boldsymbol{D}}\mathbf{=0.02.}$ | |

| a) | b) |
| --- | --- |
| 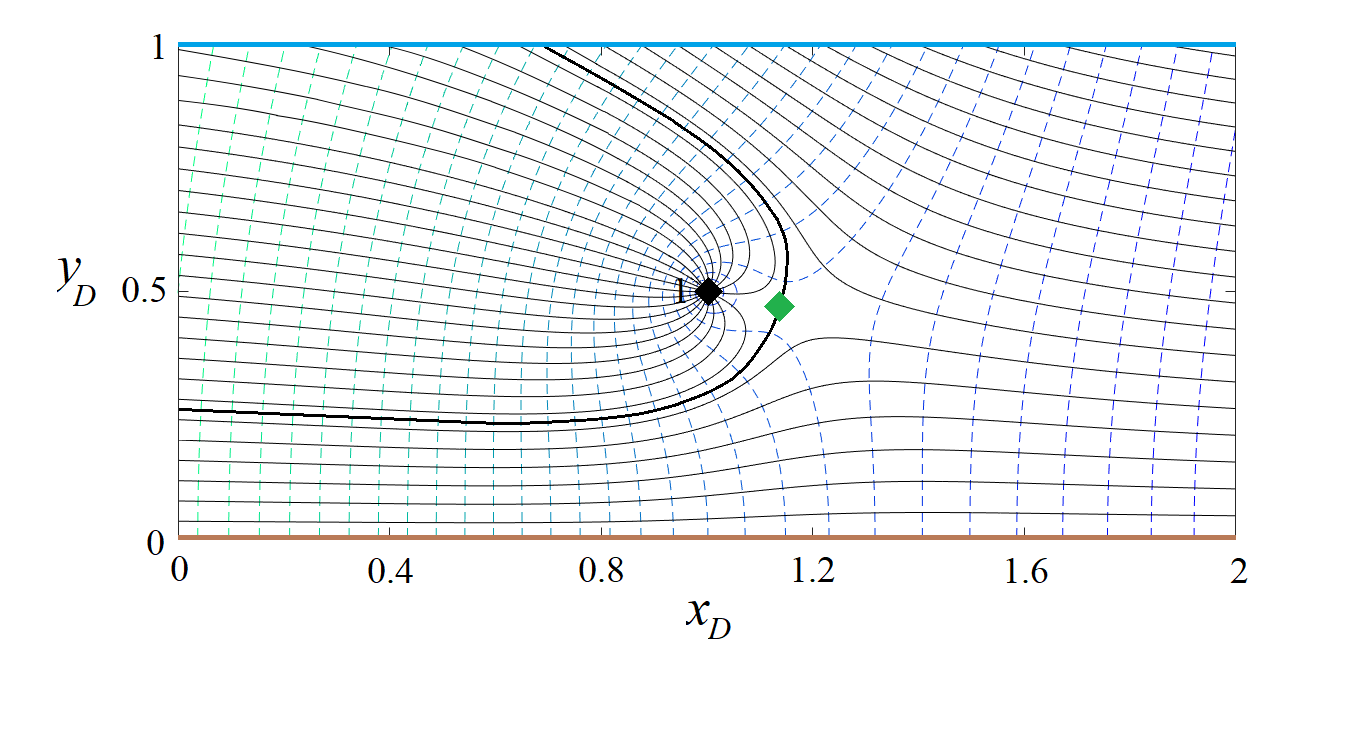 | 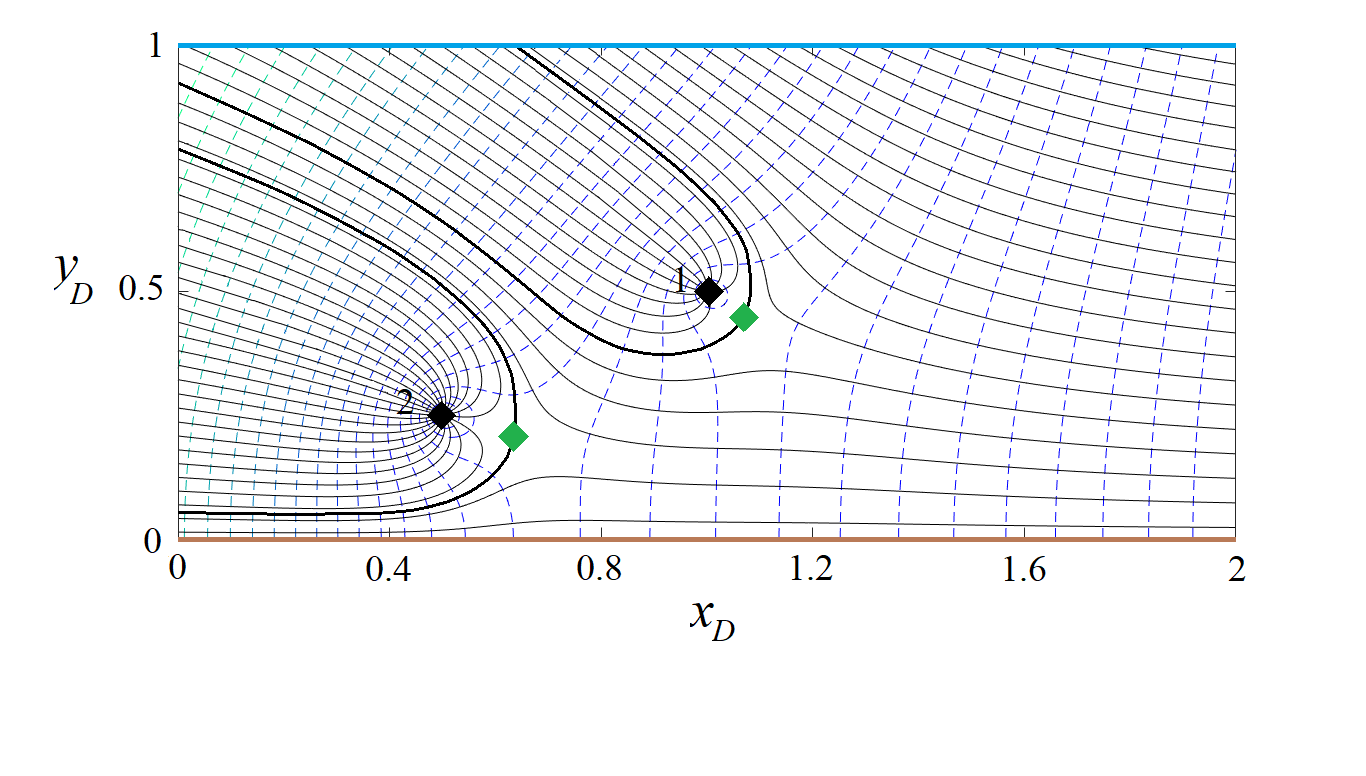 |
| c) | d) |
| 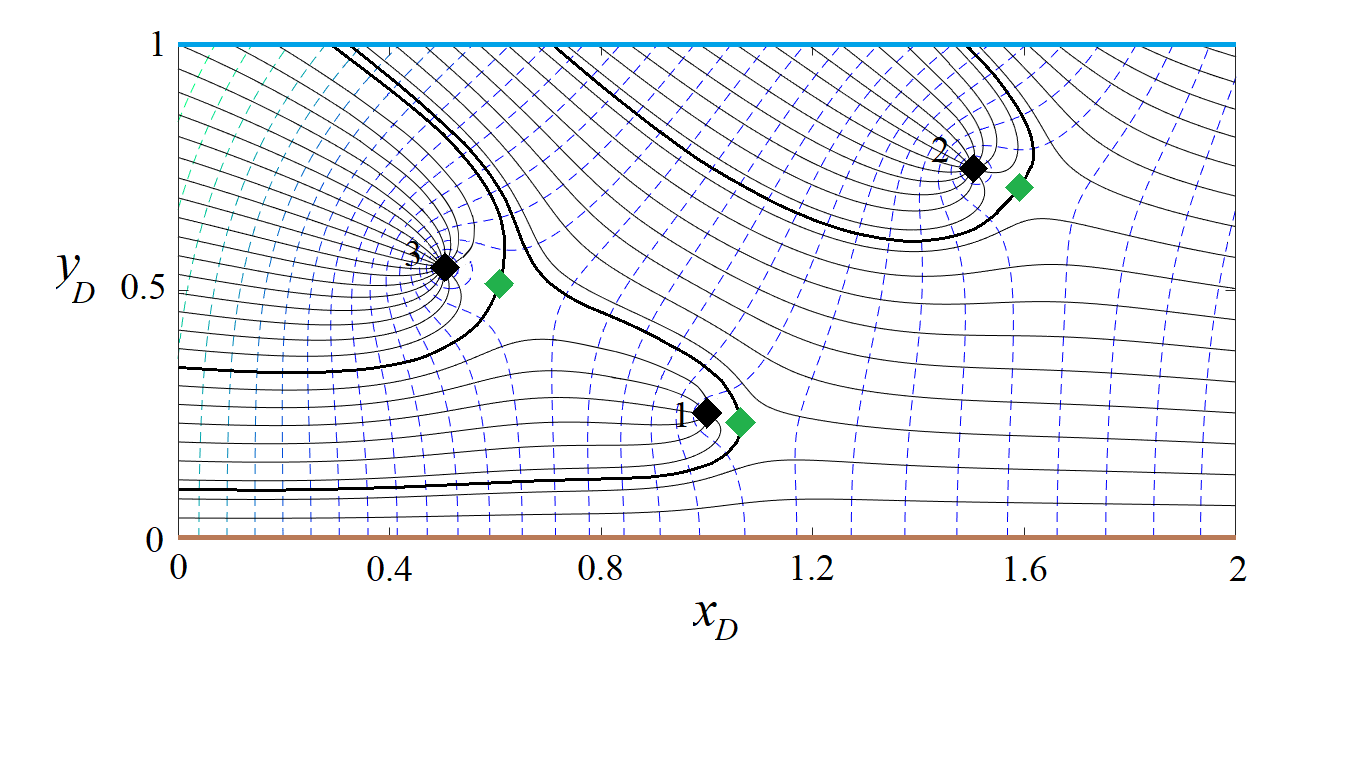 | 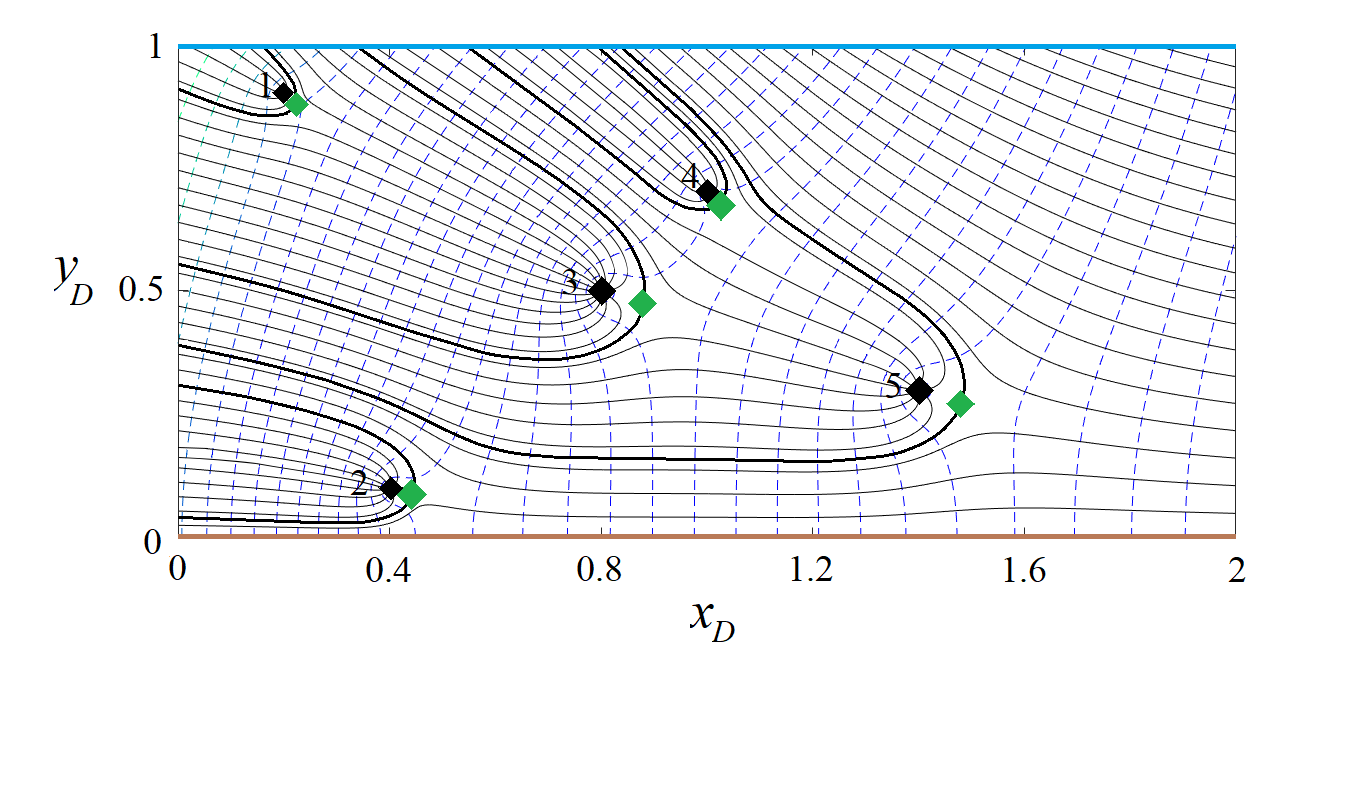 |
| **Figure E. Velocity potential and stream function for one (a), two (b), three (c) and five (d) wells in a strip-shaped aquifer with inflow-barrier boundary conditions with** $\boldsymbol{\beta}\mathbf{=0}$**and** $\boldsymbol{q}_{\boldsymbol{0}\boldsymbol{D}}\mathbf{=0.02}$**.** | |

| a) | b) |
| --- | --- |
| 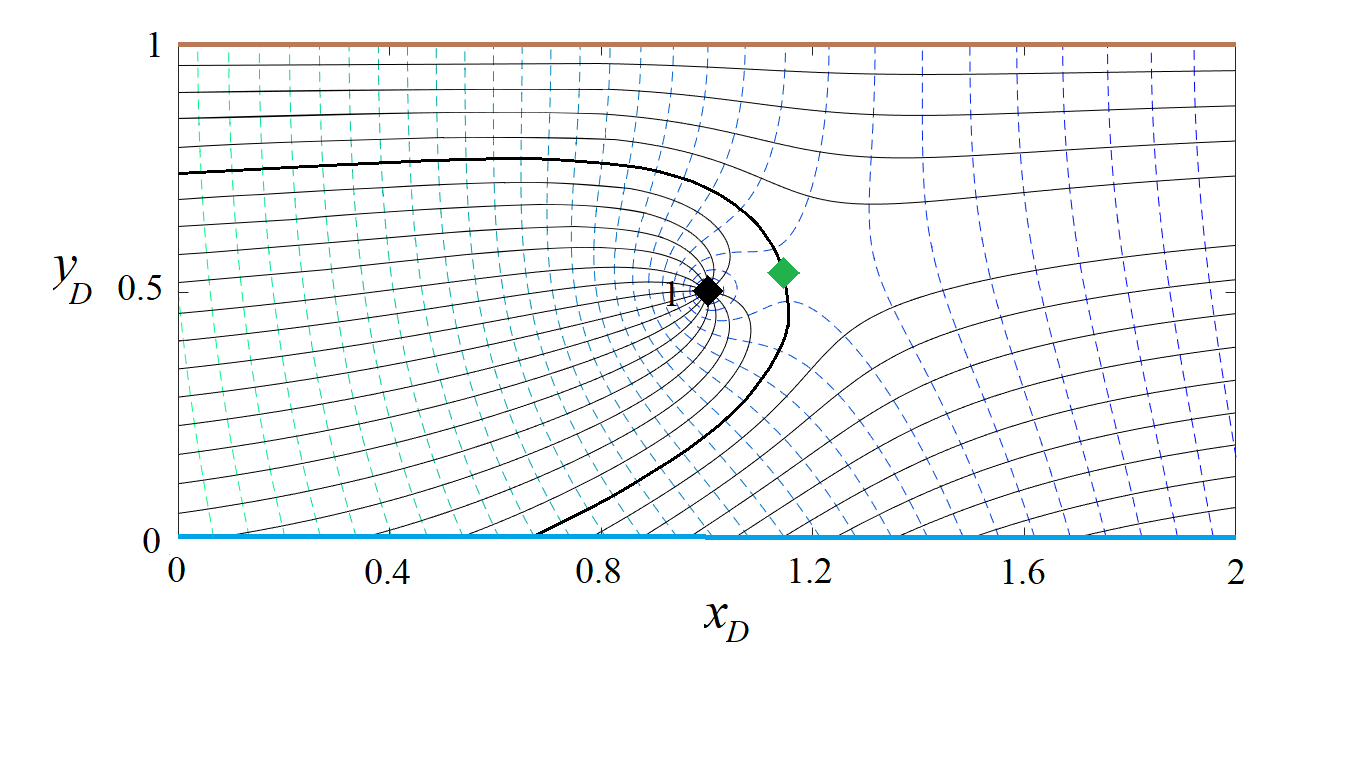 | 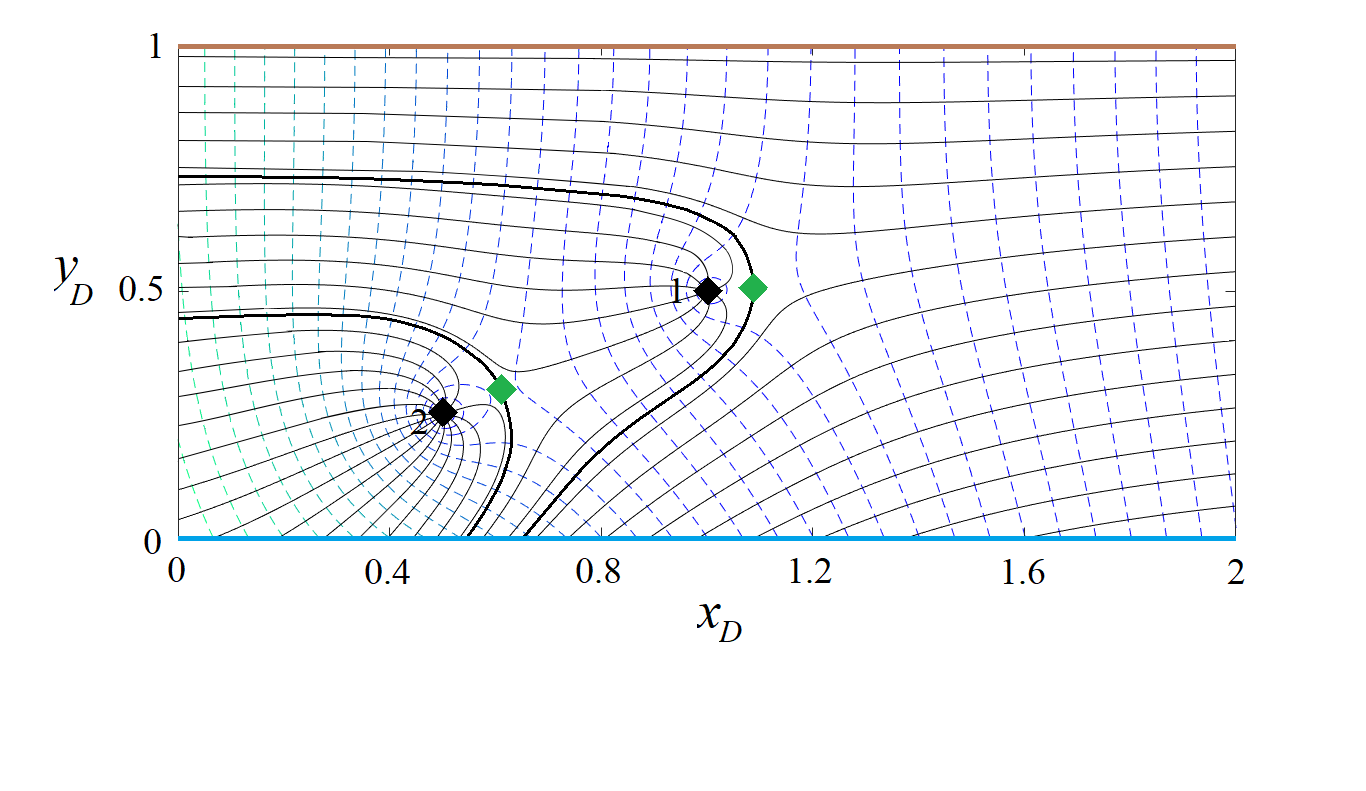 |
| c) | d) |
| 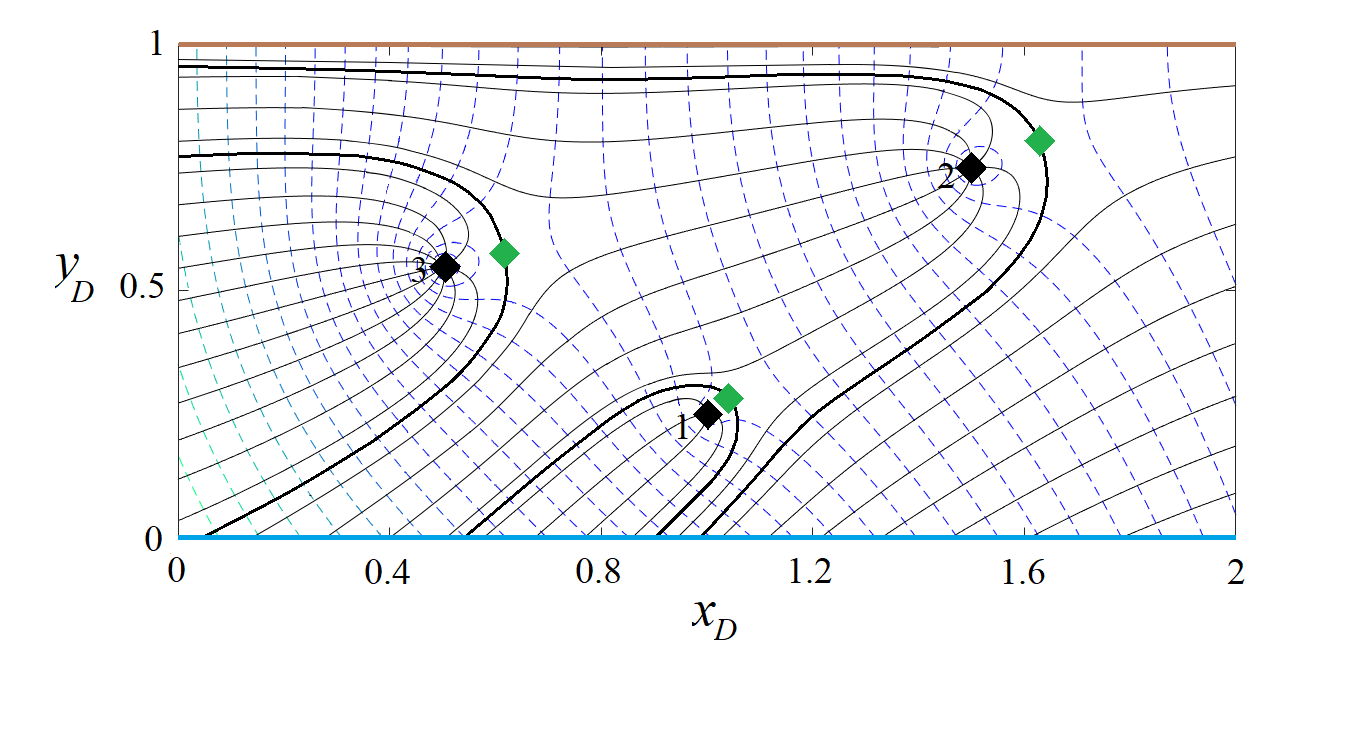 | 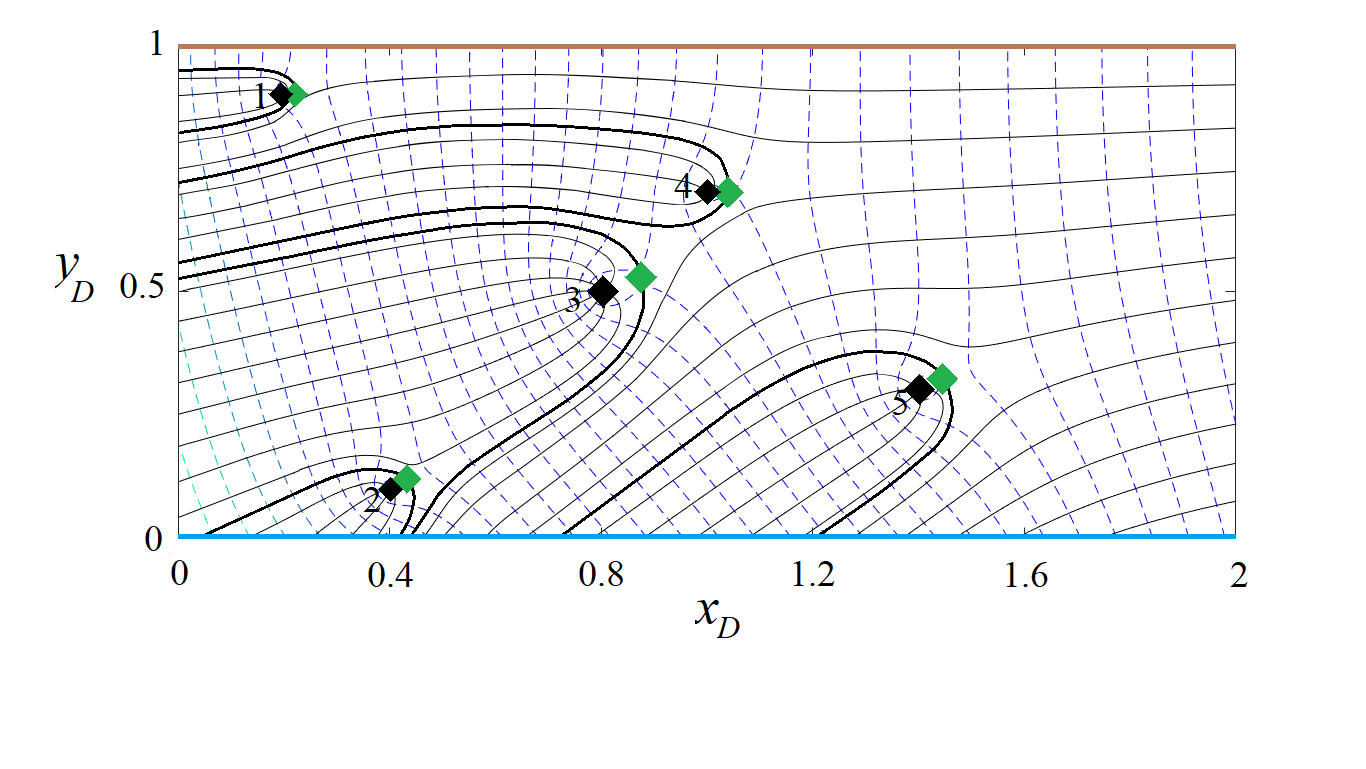 |
| **Figure F. Velocity potential and stream function for one (a), two (b), three (c) and five (d) wells in a strip-shaped aquifer for barrier-inflow boundaries with** $\boldsymbol{\beta}\mathbf{=0}$**and** $\boldsymbol{q}_{\boldsymbol{0}\boldsymbol{D}}\mathbf{=0.02.}$ | |

| a) | b) |
| --- | --- |
| 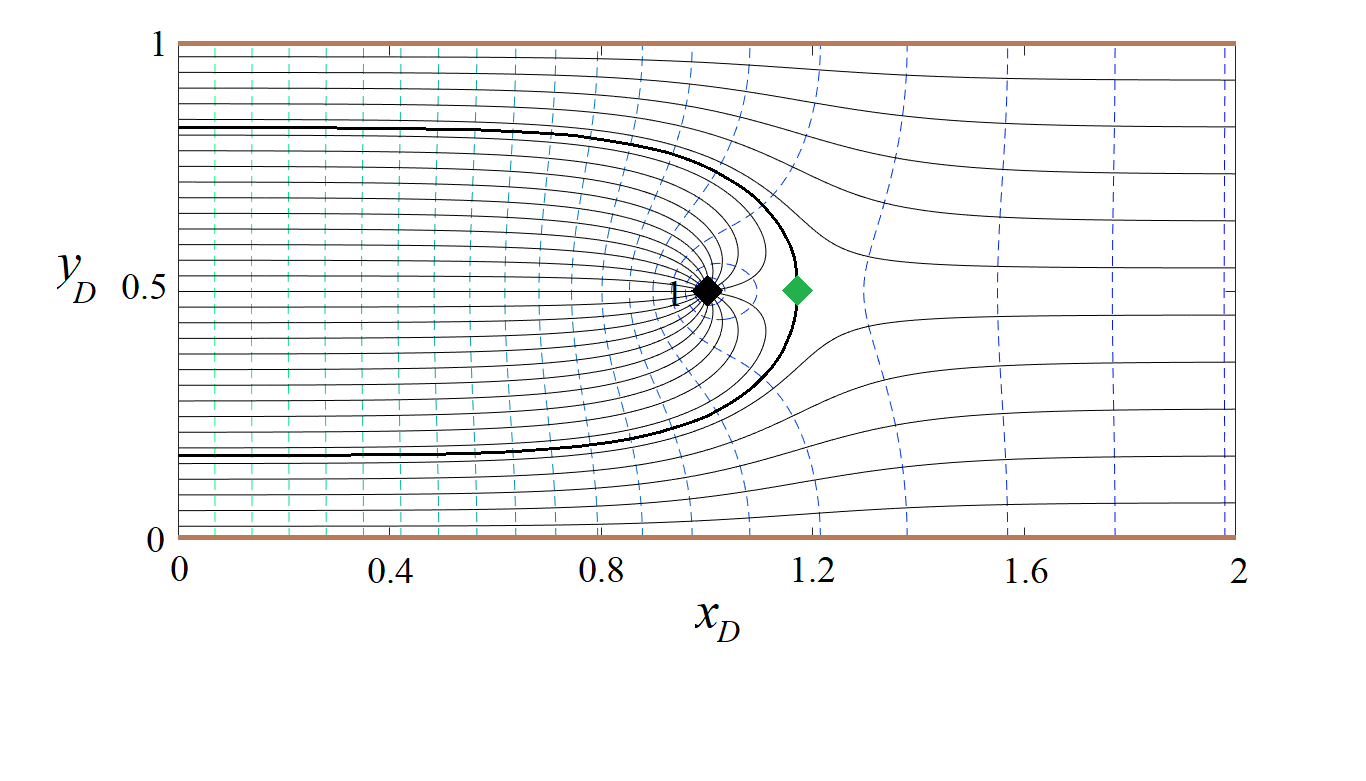 | 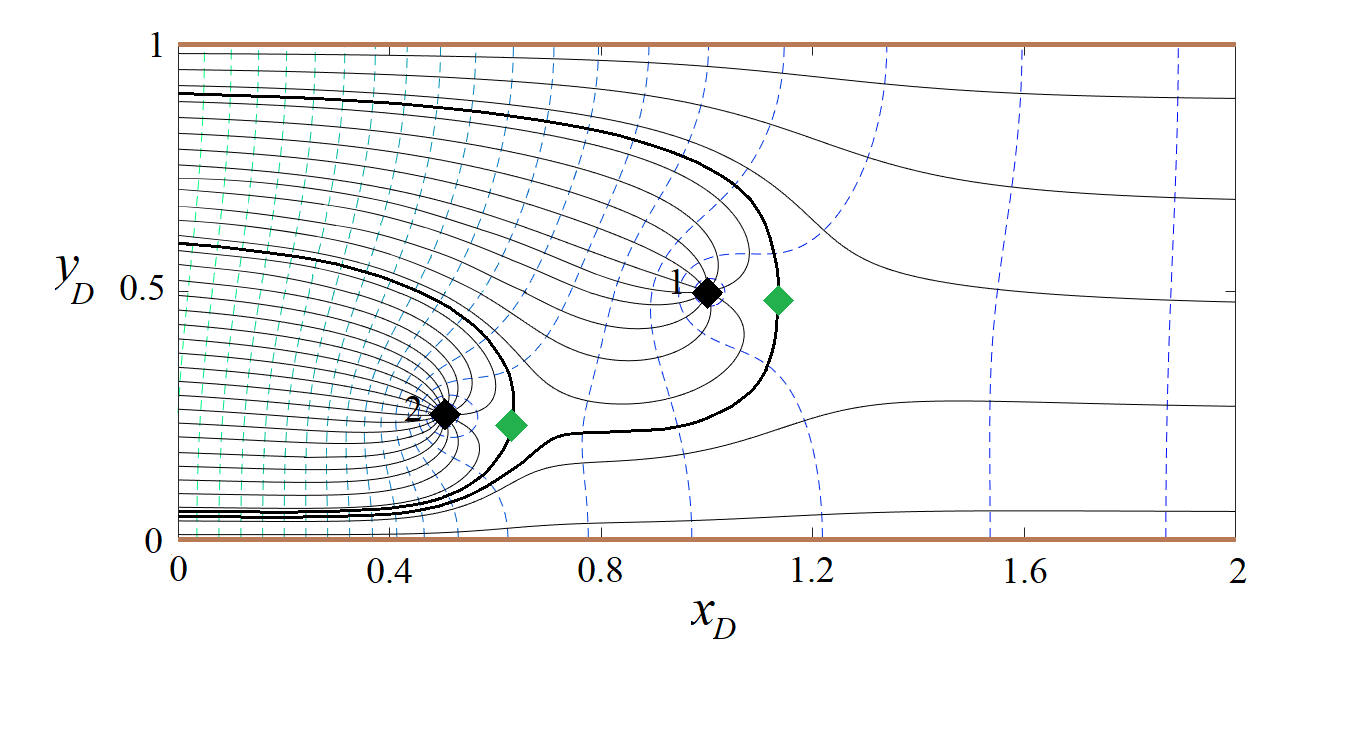 |
| c) | d) |
| 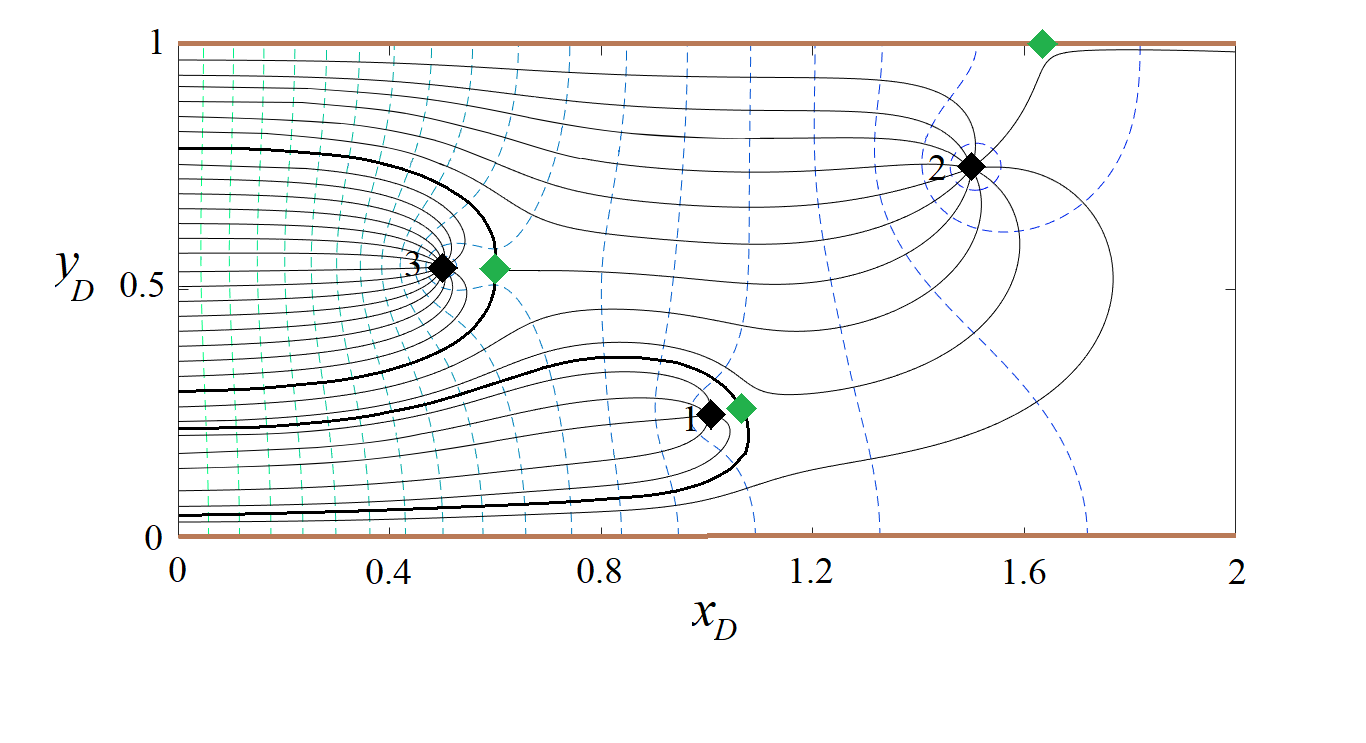 | 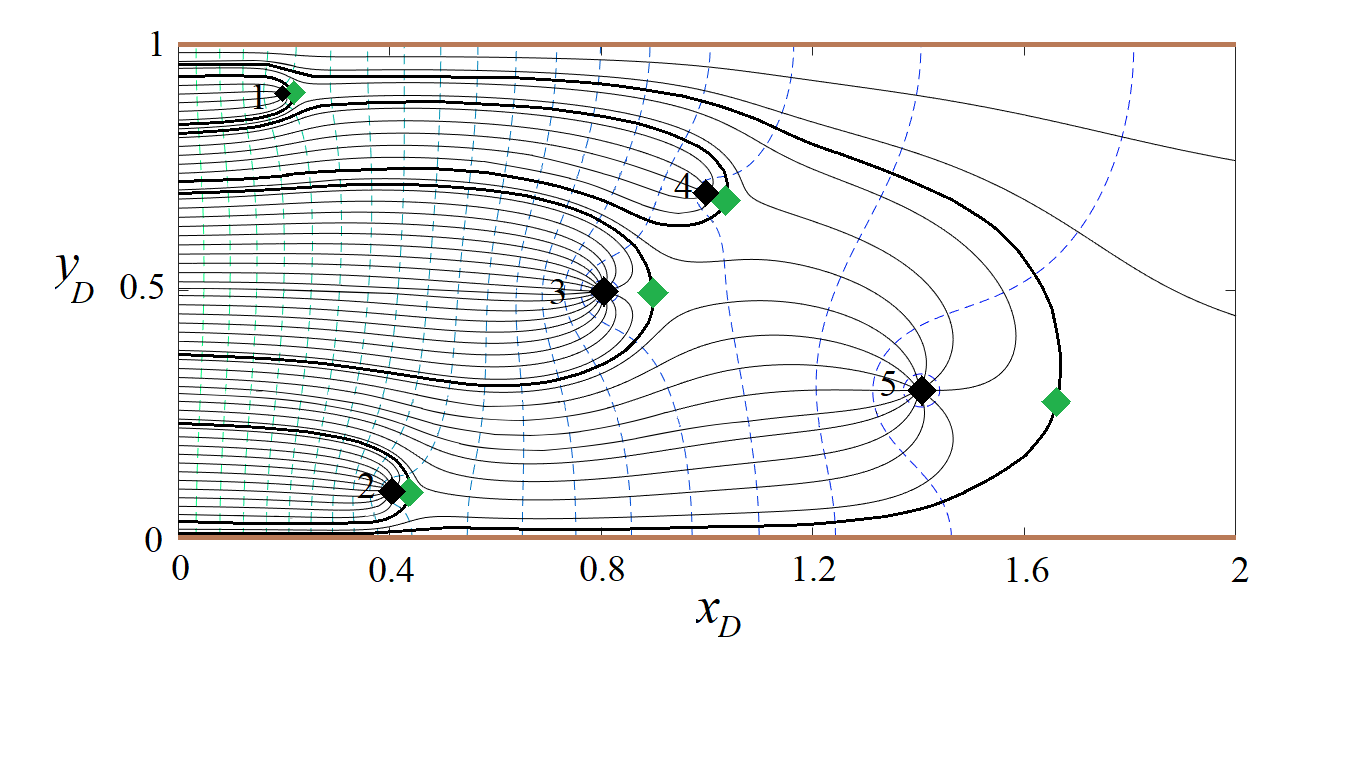 |
| **Figure G. Velocity potential and stream function for one (a), two (b), three (c) and five (d) wells in a strip-shaped aquifer for barrier-barrier boundary conditions with** $\boldsymbol{\beta}\mathbf{=0}$ **and** $\boldsymbol{q}_{\boldsymbol{0}\boldsymbol{D}}\mathbf{=0.02}$**.** | |

| a) | b) |
| --- | --- |
| 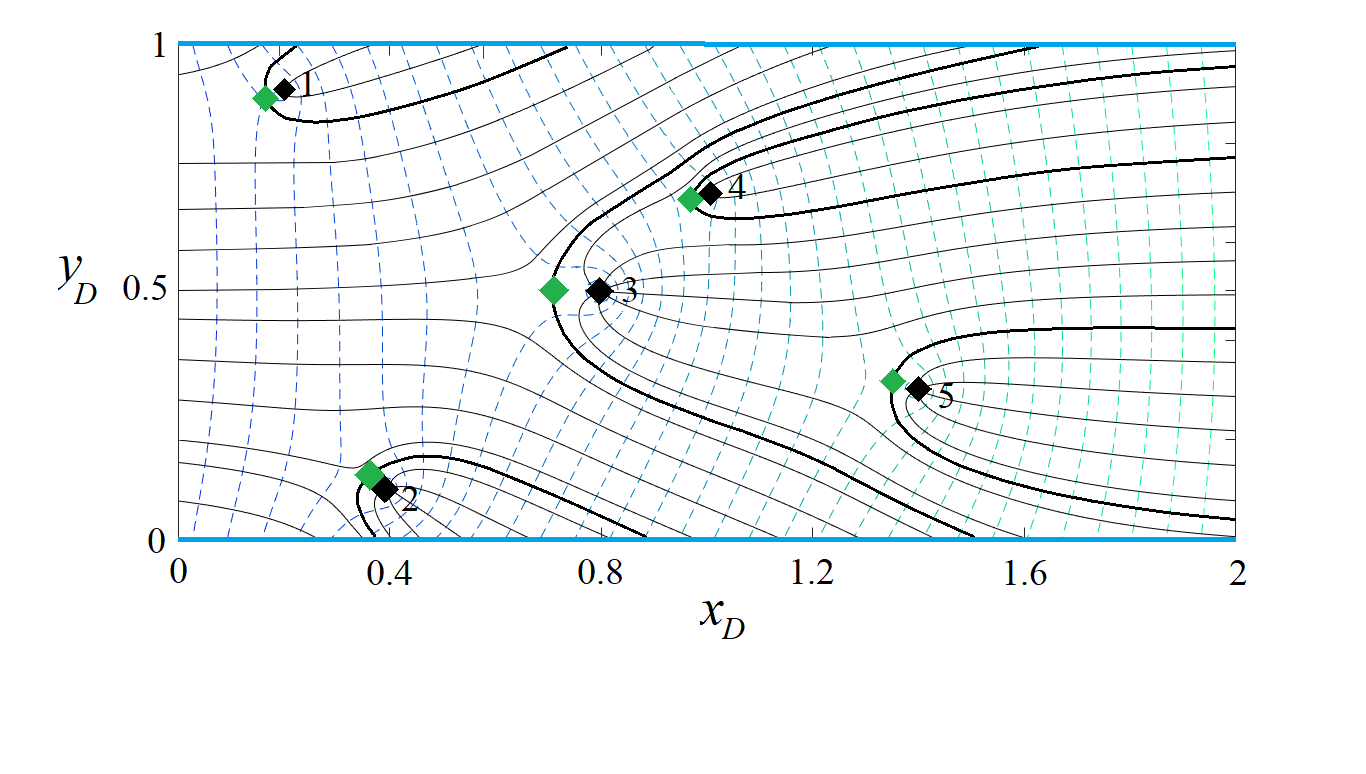 | 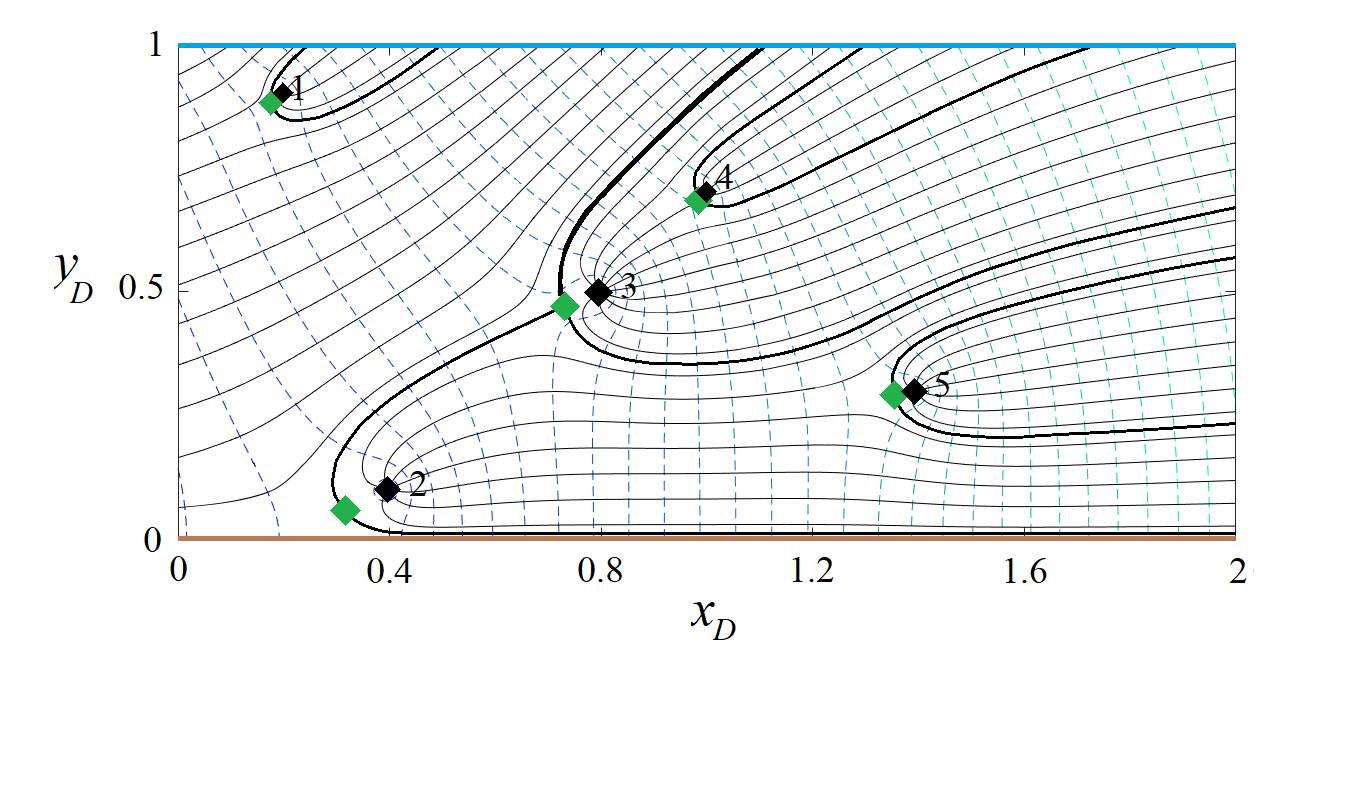 |
| c) | d) |
| 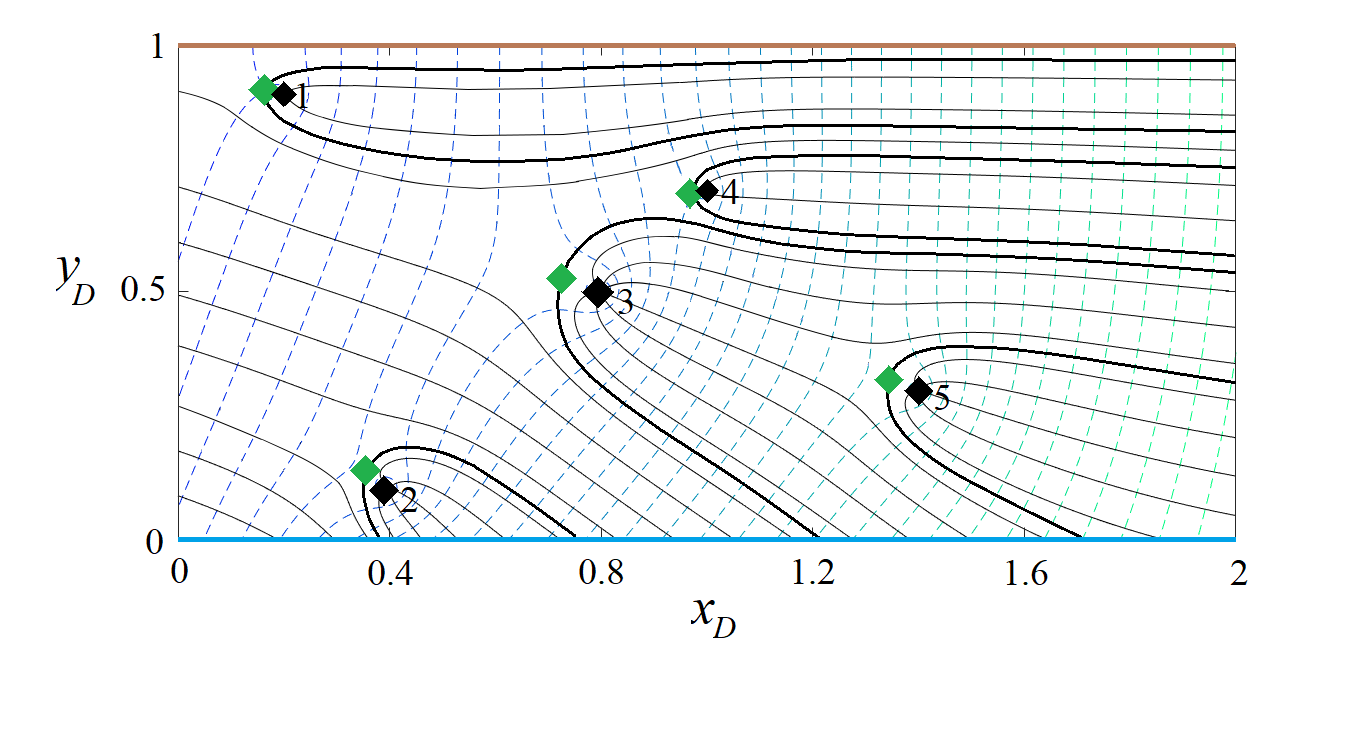 | 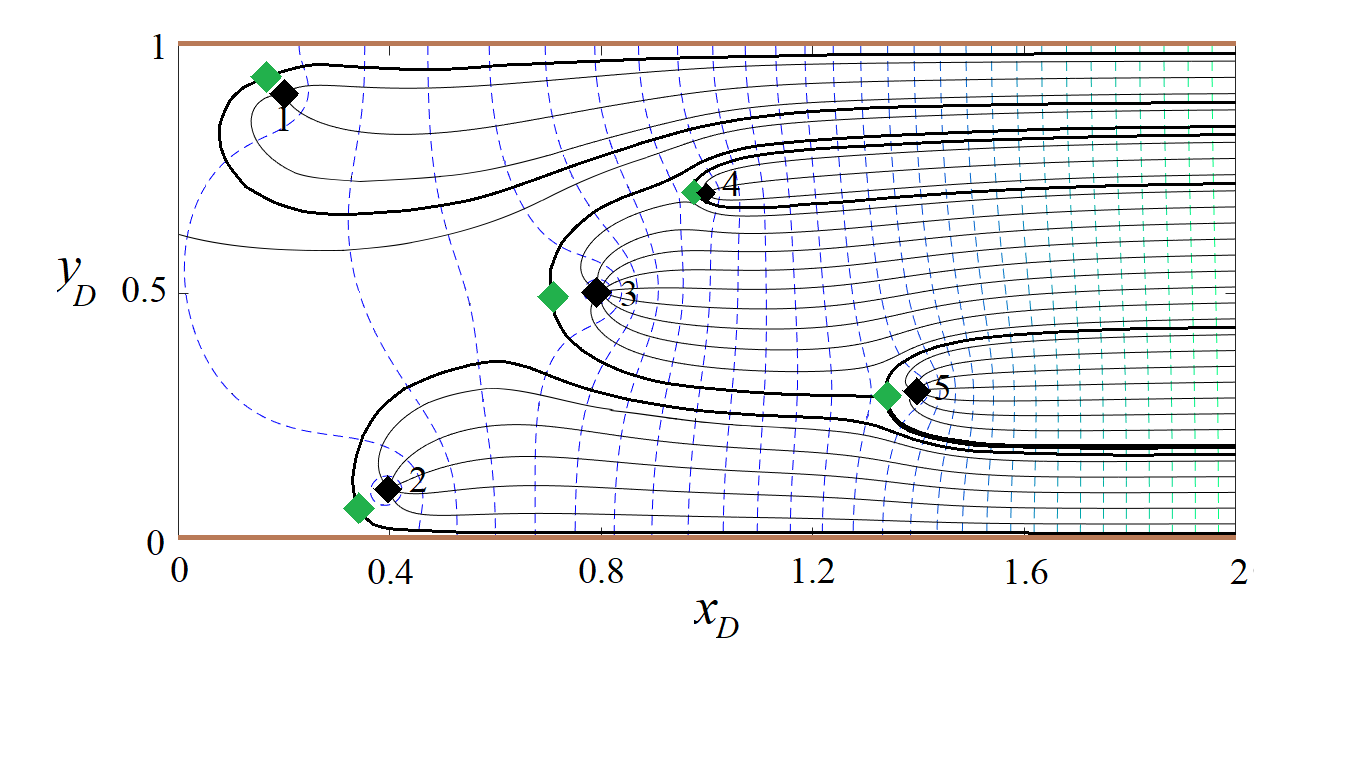 |
| **Figure H. Velocity potential and stream lines when** $\boldsymbol{\beta=\pi}$ **in strip-shaped aquifers with five extraction wells**. | |

**Figure I. Figure 10 generated numerically.**
